# An RNAi-mediated screen identifies novel targets for next-generation antiepileptic drugs based on increased expression of the homeostatic regulator pumilio

**DOI:** 10.1080/01677063.2018.1465570

**Published:** 2018-05-02

**Authors:** Wei-Hsiang Lin, Miaomiao He, Yuen Ngan Fan, Richard A. Baines

**Affiliations:** Division of Neuroscience and Experimental Psychology, School of Biological Sciences, Faculty of Biology, Medicine and Health, University of Manchester, Manchester Academic Health Science Centre, Manchester, UK

**Keywords:** *Drosophila*, epilepsy, neuronal homeostasis, voltage-gated sodium current

## Abstract

Despite availability of a diverse range of anti-epileptic drugs (AEDs), only about two-thirds of epilepsy patients respond well to drug treatment. Thus, novel targets are required to catalyse the design of next-generation AEDs. Manipulation of neuron firing-rate homoeostasis, through enhancing Pumilio (Pum) activity, has been shown to be potently anticonvulsant in *Drosophila*. In this study, we performed a genome-wide RNAi screen in S2R + cells, using a luciferase-based dPum activity reporter and identified 1166 genes involved in dPum regulation. Of these genes, we focused on 699 genes that, on knock-down, potentiate dPum activity/expression. Of this subgroup, 101 genes are activity-dependent based on comparison with genes previously identified as activity-dependent by RNA-sequencing. Functional cluster analysis shows these genes are enriched in pathways involved in DNA damage, regulation of cell cycle and proteasomal protein catabolism. To test for anticonvulsant activity, we utilised an RNA-interference approach *in vivo*. RNAi-mediated knockdown showed that 57/101 genes (61%) are sufficient to significantly reduce seizure duration in the characterized seizure mutant, *para^bss^*. We further show that chemical inhibitors of protein products of some of the genes targeted are similarly anticonvulsant. Finally, to establish whether the anticonvulsant activity of identified compounds results from increased *dpum* transcription, we performed a luciferase-based assay to monitor *dpum* promoter activity. Third instar larvae exposed to sodium fluoride, gemcitabine, metformin, bestatin, WP1066 or valproic acid all showed increased *dpum* promoter activity. Thus, this study validates Pum as a favourable target for AED design and, moreover, identifies a number of lead compounds capable of increasing the expression of this homeostatic regulator.

## Introduction

Epilepsy is a common neurological disorder characterized by recurrent and unprovoked seizures. The causes of epilepsy are varied with, perhaps, the majority being due to gene mutations. To date upwards of 500 genetic loci have been identified as contributory to epilepsy (Noebels, 2015). However, while the primary cause of epilepsy varies, a principle commonality underlying seizure generation is neuronal hyperexcitability and/or synchronicity of activity. A hyperactivate state invariably occurs due to either increased excitatory or decreased inhibitory synaptic neurotransmission, which itself is often caused by altered kinetics of voltage-gated ion channels in either excitatory or inhibitory neurons (Bradford, 1995; Lin and Baines, 2015; Turrigiano and Nelson, 1998). Additional changes in either intra- or extra-cellular ion concentrations can also lead to altered signalling through wild-type ion channels. For example, mutations in the K^+^/Cl^−^ co-transporter NKCC1 can result in GABA-induced excitation instead of inhibition (Lykke *et al*., 2016).

It is not surprising that a majority of AEDs target ion channels or neurotransmitter signalling to limit neuronal hyperexcitability. Primary targets include voltage-gated Na^+^ channels (e.g. phenytoin and carbamazepine), GABA-signalling, (e.g. gabapentin, vigabatrin, tiagabine) and synaptic vesicle protein 2 A (levetiracetam) (Klitgaard *et al*., 2016; Lason, Chlebicka, & Rejdak, 2013). However, despite the availability of a wide range of AEDs, only about two-thirds of epilepsy patients respond to drug treatment. Because of this, there is a clear and currently unmet clinical need for next-generation AEDs that modify novel targets. Exploitation of model organisms such as *Caenorhabditis elegans*, *Drosophila melanogaster* and *Danio rerio* offer the possibility to accelerate the identification of novel targets. The high degree of conservation in CNS development and function across animals makes these ‘simpler’ models highly attractive for drug development. In addition to being suited for high-throughput screening, seizures can be induced in these models using the same methods that prevail in rodents; proconvulsants, electroshock or genetic modification of homologous genes (recently reviewed in: Baines, Giachello, & Lin, 2017; Copmans, Siekierska, & de Witte, 2017; Takayanagi-Kiya and Jin, 2017).

We have recently reported a novel approach to control seizure behaviour in *Drosophila* which, initial studies suggest may be applicable to humans. In brief, pan-neuronal up-regulation of *dpum* is sufficient to dramatically reduce seizure duration in a range of bang-sensitive (bs, seizure) mutations (specifically, *para^bs^* ^s^, *easilyshocked* and *slamdance*) (Lin, Giachello, & Baines, 2017). Pum is a key component of a neuronal homeostatic mechanism (termed firing-rate homoeostasis) that maintains action potential firing within physiologically-appropriate limits (Mee, Pym, Moffat, & Baines, 2004; Muraro *et al*., 2008). Pum is a member of the Pum and FBF (PuF) RNA-binding protein family and is evolutionarily conserved in many species including flies and mammals (Wickens, Bernstein, Kimble, & Parker, 2002; Zamore, Williamson, & Lehmann, 1997). By binding an eight nucleotide sequence in mRNA (UGUA(A/U/C)AUA), termed a Pum Response Element (PRE), Pum represses translation and reduces protein synthesis (Arvola, Weidmann, Tanaka Hall, & Goldstrohm, 2017; Wharton, Sonoda, Lee, Patterson, & Murata, 1998; Wreden, Verrotti, Schisa, Lieberfarb, & Strickland, 1997). Pum activity is regulated by neuronal depolarization: increased synaptic excitation elevates Pum expression and increased translational repression of *voltage-gated sodium channel* (*Na_v_*) transcripts. This is sufficient to reduce neuron Na^+^ current (I_Na_) and action potential firing (Mee, *et al*., 2004; Muraro, *et al*., 2008). An identical mechanism, mediated by the homologue Pum2, acts to repress translation of mammalian *Na_v_* mRNA; specifically *scn1A* and *scn8A* (Driscoll, Muraro, He, & Baines, 2013; Vessey *et al*., 2006). Indeed, it is now widely believed that without homoeostatic regulation of neuron excitability, chronic changes in levels of synaptic excitation would destabilise neural circuits leading to an imbalance in the excitation-inhibition balance (Giachello and Baines, 2017). In this regard, it is intriguing that Pum expression is down-regulated in fly seizure mutants, rat induced-seizure models and in human temporal lobe epilepsy (Lin, *et al*., 2017; Wu *et al*., 2015). *Pum2* knockout mice also show spontaneous seizures (Follwaczny *et al*., 2017; Siemen, Colas, Heller, Brustle, & Pera, 2011). Thus, neuronal homeostasis and, specifically Pum, may offer an attractive route for the development of next-generation AEDs.

We have recently reported a luciferase-based reporter of dPum activity and screened an FDA-approved drug library to identify compounds that promote the activity of this homeostatic regulator (Lin, *et al*., 2017). This screen identified, amongst other compounds, avobenzone. Our follow-on studies indicate this compound promotes transcription of *dpum* and increased dPum protein. Moreover, this compound has potent anticonvulsive properties when fed to bs mutant *Drosophila*. In this present study, we expand our screening to incorporate a genome-wide RNAi library. We identify 699 RNAi’s that are sufficient to potentiate dPum activity. A comparison of these 699 genes with activity-dependent genes, identified through an RNA-sequencing approach (Lin, *et al*., 2017), shows that 101 genes are also regulated by synaptic activity. The protein products of these 101 genes may prove to be favourable targets for drug-mediated inhibition to better control epilepsy. To show proof-of-principle, we express RNAi targeted to these genes in the *para^bss^* seizure mutant background and report that 57 significantly reduce seizure duration. We validate, where possible, anticonvulsant effects through feeding of chemical inhibitors for the respective gene protein products.

## Materials and methods

### Luciferase-based gene cassettes report dPum activity in S2R + cells

A *firefly-*PRE reporter gene, containing two Pum Response Elements (PRE^1^ and PRE^2^) (Gupta *et al*., 2009), cloned from a region of the *hunchback* 3′UTR (NM_169233.2, 2390–2650), was used as described in Lin, *et al*., 2017. A *renilla* luciferase reporter, lacking the PRE motifs, was used as a reference to report expression efficiency.

### Genome-wide double-stranded RNA library screen

Insect S2 cells, derived from a primary culture of late stage (20–24 h old) *Drosophila* (Oregon-R) embryos (Schneider, 1972), are widely used to carry out large-scale functional screens (Boutros *et al*., 2004; Kleino *et al*., 2005; Ramet, Manfruelli, Pearson, Mathey-Prevot, & Ezekowitz, 2002). The S2R + subtype, used in this study, differs in the expression of the membrane receptor *Drosophila frizzled 2* (*Dfz2*) (Yanagawa, Lee, & Ishimoto, 1998), making them more adherent than S2 cells and readily attach and spread to tissue culture plastic and glass. S2R + cells (1.5 × 10^4^ cells in 15 μl of Schneider's *Drosophila* Medium, Gibco^TM^) were treated with 250 ng of double-stranded RNA (∼21,000 double-stranded RNAs, ∼98.8% coverage, covering ∼14,000 protein encoding genes and ∼1000 noncoding genes on 53 × 384 well plates) for 48 h, followed by co-transfection (Effectene^®^, QIAGEN) with *firefly*-PRE and *renilla* luciferase reporters (10 ng each) (Lin *et al*., 2017) for a further 48 h. The transfection procedure is as described in the manufacturer’s instructions (QIAGEN). S2R + cells were lysed with 0.35% Triton^TM^ X-100 in BL buffer (50 mM HEPES, 0.5 mM EDTA, 0.36 mM phenylacetic acid and 0.07 mM oxalic acid) and D-Luciferin (0.46 mM, Molecular Probes) was added to measure firefly luciferase activity. This was followed by the addition of coelenterazine-h (3 mM, Promega) to measure *renilla* luciferase activity. A Varioskan^®^ flash plate reader (Thermo Scientific) was used to measure luminescence.

### Bioinformatics

Functional cluster analysis of 1166 *dpum* regulators was carried out using DAVID 6.8 software (the Database for Annotation, Visualization, and Integrated Discovery) (https://david.ncifcrf.gov) (Huang da, Sherman, & Lempicki, 2009a, 2009b). Sets of genes were uploaded using FLYBASE gene IDs. The *p* values for enrichment of genes in biological mechanisms were evaluated by Benjamini correction, and values less than 0.05 were considered significant. The molecular interaction networks of 101 *dpum* activity-dependent regulators were investigated using Cytoscape v. 3.5.1 software (http://www.cytoscape.org/) (Saito *et al*., 2012; Shannon *et al*., 2003). The networks of gene relationships were based on the *Drosophila melanogaster* gene annotation databases. Results were visualized using ClueGO v. 2.5.0 (Bindea *et al*., 2009), CluePedia v. 1.5.0 (Bindea, Galon, & Mlecnik, 2013), and Cytoscape plug-in apps. Selection criteria was at least three genes per node with a minimum of 4% of the associated genes from all uploaded genes in one node. The threshold of pathway network connectivity (Kappa score) was 0.4 and pathways with *p* values ≤.05 are shown.

### Validation of RNAi knock down efficacy by quantitative PCR

Quantitative RT-PCR was performed using a SYBR Green I real-time PCR method (Roche, LightCycler^®^ 480 SYBR Green I Master, Mannheim, Germany) as described in Lin, He, & Baines (2015). RNA was extracted from 20 male adult heads using the RNeasy micro kit (QIAGEN). Primer sequences (5′–3′) are listed in Supplementary Table 1. Relative gene expression was calculated using 2^−ΔCt^, where ΔCt was determined by subtracting the average *actin-5C* Ct value for each gene measured.

### Behavioural screening on a bang sensitive mutant, para^bss^

Seizure duration in adult flies is determined as described in Lin *et al*. (2015). In brief, 20 virgin females of *para^bss^*;GAL4*^Cha(19B)^* (expressing in all cholinergic neurons) were crossed to five UAS-RNAi males. Only *para^bss^*/*Y;*GAL4*^Cha(19B)^/UAS-RNAi* hemizygous male progeny were used for behavioural screening. Flies (two to three days old) were tested at least one day after collection to ensure total recovery from CO_2_-anaesthesia. Flies were transferred to an empty vial (10 per vial) and left to recover for 30 min, before being exposed to mechanical shock by vortexing the vial at maximum speed for 10 s. Recovery Time (RT) was calculated from the average time taken for all 10 flies to recover from paralysis to standing. At least five replicates were performed for each RNAi line. Values were compared with control flies (*para^bss^/Y*;GAL4*^Cha(19B)^/+*).

### Fly stocks

Flies were maintained on standard cornmeal medium at 25 °C. *para^bss^* were gifts from Dr. Kevin O’Dell (University of Glasgow). The *elav*-GAL4^C155^ (stock no. 458) was obtained from Bloomington and UAS-RNAi lines were obtained from the Vienna *Drosophila* Resource Center. *Para^bss^*;GAL4*^Cha(19B)^*was derived by crossing *para^bss^* with GAL4*^Cha(19B)^* (a gift from Dr. Paul Salvaterra, City of Hope, USA).

### Drug feeding and seizure behaviour test in 3rd instar larvae

Wall-climbing 3rd instar larvae were subjected to an electric shock to induce seizure, with or without previous feeding of drug, as described previously (Marley and Baines, 2011). For drug feeding studies, eggs were laid on food containing drug and larvae were raised until wall-climbing 3rd instar. Concentration of the drugs used are as follows and the most effective concentration shown in Table 2 is underlined: SB203580 (2.6 and 13 µM, S1076, Selleckchem), Losmapimod (2.6, 13 and 26 µM, S7215, Selleckchem), sodium fluoride (1.2 and 2.4 mM, S7920, Sigma-Aldrich), gemcitabine (3.3, 16.5 and 165 µM, G6423, Sigma-Aldrich), Metformin (1.2, 2.4 and 3.6 mM, PHR1084, Sigma-Aldrich), bestatin (81 and 162 µM, J61106.MC, Alfa Aesar), WP1066 (140 and 281 µM, 573097, Merck), valproic acid (0.6, 1.2 and 2.4 mM, P4543, Sigma-Aldrich) and phenytoin (1.6 mM, D4505, Sigma-Aldrich). In response to electroshock, larvae undergo a transitory paralysis during which they tonically contract and, occasionally, spasm (see (Marley and Baines, 2011) for details on seizure behavior). Recovery time reported represents the average time for larvae to resume normal crawling behaviour and at least 30 larvae were tested for each chemical inhibitor treatment.

### Luciferase-based promoter assay

Luciferase activity in 3rd instar larvae was measured using the Promega Steady-Glo Luciferase Assay Kit. Briefly, a *dpum* promoter-GAL4 line (containing a 578-bp region upstream of the *dpum* transcription start site) was crossed to attP24 UAS-*luciferase* flies (Markstein, Pitsouli, Villalta, Celniker, & Perrimon, 2008). Three larvae were collected in 200 µl Promega Glo Lysis buffer for each sample, and at least 5 independent samples collected for each genotype. Larvae were homogenized, incubated at room temperature for 10 min, centrifuged for 5 min, and supernatant was transferred to a new tube. For luciferase assays, 30 µl of each sample was transferred to a well of a white-walled 96-well plate at room temperature, 30 µl Promega Luciferase reagent was added to each well and plates were incubated in the dark for 10 min. Luminescence was measured with a GENios plate reader (TECAN). The obtained values were normalized to total protein concentration, measured using the Bradford protein assay (Bio-rad).

#### Statistics

Normality of the data were checked using the D' Agostino-Pearson omnibus test before parametric statistical tests were applied. Statistical significance between group means was assessed using either a Student’s *t*-test (where a single experimental group is compared to a single control group) or ANOVA followed by the Bonferroni’s *post hoc* test (multiple experimental groups). Data shown is mean ± standard deviation (s.d.).

## Results

### A genome-wide RNAi screen to identify potential regulators of pumilio

**Figure 1.**
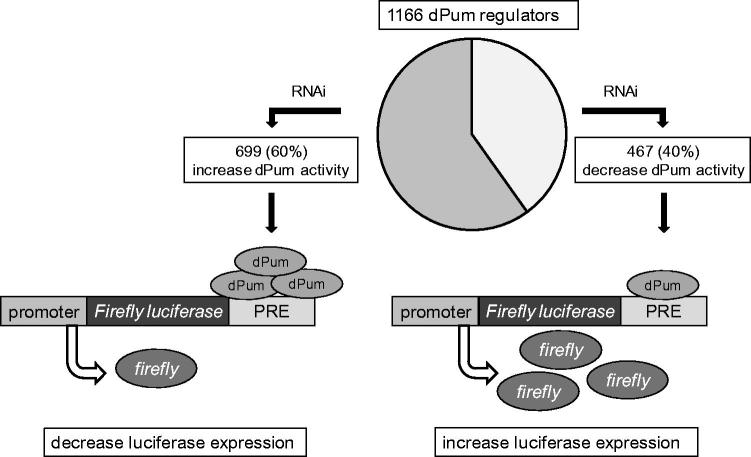
A genome-wide RNAi screen identified 1166 *Drosophila* (dPum) regulators. Using a dPum activity luciferase-reporter to screen a genome-wide RNAi library identified 699 and 467 gene knockdowns that increase or supress dPum activity, respectively. The dPum activity reporter used is the *firefly*-luciferase gene driven by an *actin* promoter (promoter) and containing two Pum Response Elements (PRE) in its 3’ UTR. Increased dPum is sufficient, through binding to the PRE and inhibiting translation, to decrease luciferase expression. Conversely, decreased dPum expression/activity results in increased luciferase expression.

Since enhanced *dpum* expression is anticonvulsant in *Drosophila* (Lin *et al*., 2017), we reasoned that identification of gene knockdowns that increase dPum activity may provide a more realistic route for the development of novel anticonvulsive compounds. This is because it is generally easier to block, rather than enhance, protein function. To identify genes capable of altering dPum activity, we screened a genome-wide RNAi library using an *actin* promoter driven *firefly*-luciferase (luc) reporter construct (FF-PRE). Increased dPum is sufficient to reduce luc activity, through binding the PRE and inhibiting translation. An identical reporter lacking PRE sites and coupled to *renilla* luc (Ren) was included to enable ratiometric determination of activity (Lin *et al*., 2017). We performed two replicates of the screen (Z-score ≥1.5 or ≤ −1.5) and identified 1191 dsRNAs (1166 genes). Among these, 467 genes enhanced FF-PRE (i.e. reduced dPum activity) on knock-down. We identified *dpum* in this group, which is predictable and serves to validate our screen methodology. The remaining 699 genes supressed FF-PRE expression when knocked-down (i.e. increased dPum activity) (Figure 1, Supplementary Table 2 for gene list). Furthermore, 25 transcripts, for example, *proteasome beta3 subunit* (CG11981) and *mediator complex subunit 10* (CG5057), were hit twice by dsRNAs (BKN28041 and BKN46221, BKN27744 and BKN46549, respectively) targeted to different regions indicative of good reproducibility of the screen. We are particularly interested in the 699 genes that, on knock-down, act as dPum activators. The protein products of these genes may act as dPum repressors, inhibition of which would be predicted to increase dPum activity (and to reduce seizure).

To further investigate the biological importance of the 1166 identified genes, we performed a gene category analysis using DAVID software (Huang da *et al*., 2009a, 2009b). Among these genes, 988 were assigned to gene identifiers recognized by the DAVID tool. Table 1 shows six significant functional annotation clusters (for more details see Supplementary Table 3). The clusters represent genes involved/associated with transcriptional regulation, the proteasome, chromatin organisation, RNA interference, DNA-directed RNA polymerase, mRNA polyadenylation, gene silencing by miRNA and the ribosome. The cluster with the highest enrichment score (8.1) included 54 genes related to transcriptional regulation.

**Table 1.** Functional cluster analysis for 1166 *Drosophila pumilio* regulators.

Functional cluster analysis			
Biological function	Enrichment score	Number of involved genes	Ontology
Transcriptional regulation	8.1	54	BP/CC/MF
proteasome	4.8	25	BP/CC/MF
chromatin organization	4.1	48	BP/CC
RNA interference	4.0	13	BP
DNA-directed RNA polymerase	2.9	11	CC/MF
mRNA polyadenylation	2.6	8	BP
gene silencing by miRNA	2.4	8	BP
ribosome	2.2	21	CC

Clustering was performed using DAVID 6.8 software.

BP: biological process; CC: cellular component; MF: molecular function.

**Table 2.** Chemical inhibitors used to validate rescue effect in *para^bss^* 3rd instar larvae.

Chemical inhibitor	Target gene	CG number	Seizure duration (%)	*p*-value
Phenytoin	–	–	52.3 ± 15.1	1.4 × 10^−16^
Gemcitabine	*ribonucleoside diphosphate reductase large subunit*	CG5371	46.0 ± 16.7	7.8 × 10^−15^
Sodium fluoride	*protein phosphatase 1 at 87B*	CG5650	52.7 ± 27.8	1.3 × 10^−9^
Metformin	*NADH dehydrogenase (ubiquinone) B12 subunit*	CG10320	55.4 ± 29.6	2.5 × 10^−6^
Losmapimod	*p38b MAPK*	CG7393	59.3 ± 23.4	4.8 × 10^−9^
SB203580			67.9 ± 26.0	4.8 × 10^−7^
WP1066	unknown gene (predicted to involve in the regulation of JAT-STAT cascade)	CG4022	61.3 ± 27.8	2.0 × 10^−6^
Valproic acid	*Histone deacetylase 3*	CG2128	71.3 ± 17.8	2.9 × 10^−8^
Bestatin	*granny smith* (contains aminopeptidase activity)	CG7340	77.6 ± 33.5	0.0015
MG-132	*proteasome beta3 subunit*	CG11981	88.0 ± 42.0	n.s
Zaprinast	*phosphodiesterase 9*	CG42276	100.9 ± 24.3	n.s
BEZ235	*meiotic 41* (aka *ATR*, belongs to the PI3/PI4-kinase family)	CG4252	94.0 ± 26.5	n.s
Thalidomide	*vihar* (contains ubiquitin protein ligase activity)	CG10682	92.5 ± 31.1	n.s
Ethosuximide	*Ca^2+^-channel protein alpha_1_ subunit T*	CG15899	92.1 ± 28.7	n.s

The wall-climbing *para^bss^* 3^rd^ instar larvae ingested chemical inhibitors were subjected to electric shock to induce seizure-like behaviour. Averaged seizure duration was normalised to vehicle control (set at 100%). Values (*n* ≥ 30, mean ± s.d.). n.s: not significant.

### Identification of activity-dependent dPum regulators

**Figure 2.**
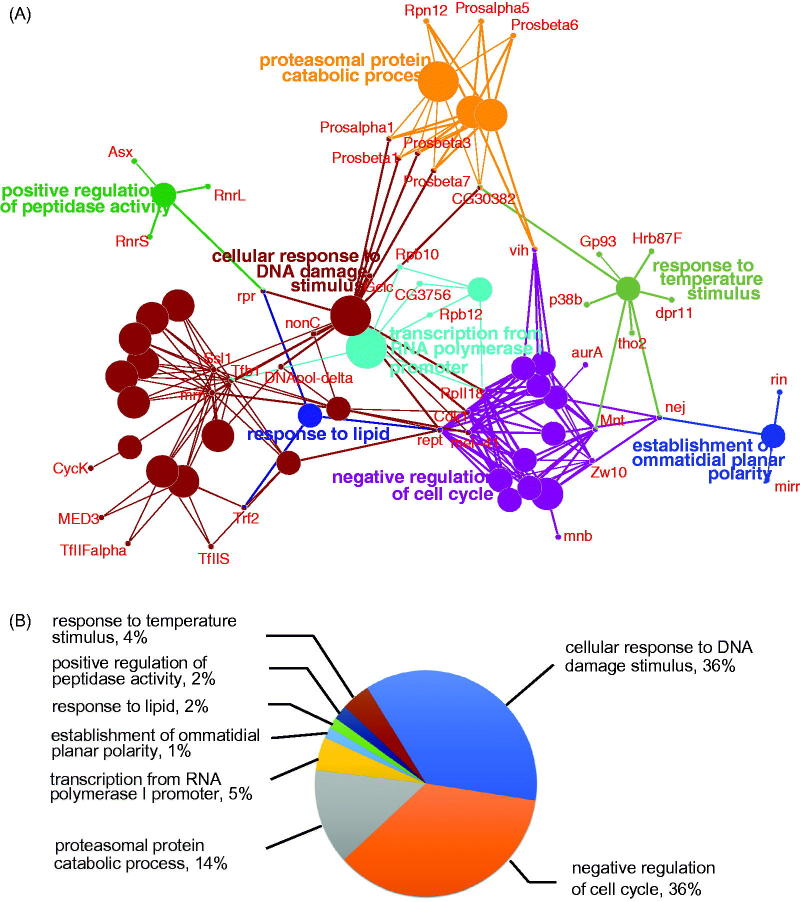
Functional cluster analysis of 101 activity-dependent dPum repressor genes. (A) The major biological functions of these 101 genes are cellular response to DNA damage, negative regulation of cell cycle, proteasomal protein catabolism, transcription from RNA polymerase I promoter, establishment of ommatidial planar polarity, response to lipid, positive regulation of peptidase activity and response to temperature. The lines represent interactions among genes. (B) Shows the distribution of identified pathway clusters.

Homeostatic control of neuron activity is itself regulated by synaptic activity (Giachello and Baines, 2017). The 699 genes, which on knock-down increase dPum activity, are of particular interest because inhibiting the gene protein products might similarly enhance dPum activity with predicted anticonvulsant effects (Lin *et al*., 2017). To refine down the number of genes to take forward, we focused on those genes which show activity-dependent transcription. We have previously reported transcriptional change in CNS between wildtype and wildtype raised on food containing the proconvulsant picrotoxin (PTX) (Lin *et al*., 2017). This identified 1685 activity dependent genes (FDR 5%). Comparison of the two gene-sets identifies 101 genes to be dPum repressors and regulated by synaptic excitation. Cytoscape bioinformatic analysis revealed that the major biological functions of these genes are: cellular response to DNA damage stimulus, negative regulation of cell cycle, proteasomal protein catabolism, transcription from RNA polymerase I promoter, establishment of ommatidial planar polarity, response to lipid, positive regulation of peptidase activity and response to temperature stimulus (Figure 2(A)). The majority of pathways identified in this analysis involve cellular response to DNA damage (36%), regulation of cell cycle (36%) and proteasomal protein catabolism (14%) (Figure 2(B)).

### Genes identified have anticonvulsant effects on knockdown

**Figure 3.**
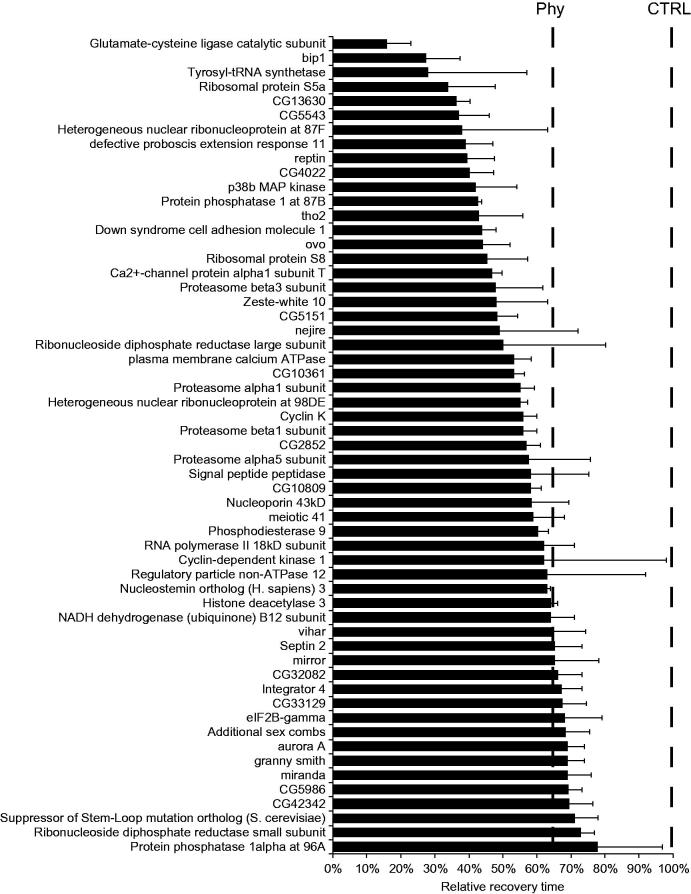
RNAi knockdown *in vivo* rescues induced-seizure duration of *para^bss^* mutant flies. Seizure mutant female flies, *para^bss^*; GAL4*^Cha(19B)^* were mated with UAS-RNAi males. F1 *para^bss^*/*Y*; GAL4*^Cha(19B)^*/UAS-RNAi hemizygous males were used for the behavioural test. Flies were subjected to a mechanical shock (10 s vortex) and recovery time measured. The recovery time of control flies (*para^bss^*/*Y*; GAL4*^Cha(19B)^*/*+*) (CTRL) was set at 100% and the relative recovery time of each gene knockdown is shown. Acutely fed phenytoin (Phy) (1.6 mM) to *para^bss^*;GAL4*^Cha(19B)^* adult flies, for 24 h, reduced seizure duration to 64.2 ± 17.0% (set vehicle control at 100%). Values (*n* = 5, mean ± s.d.) were compared by ANOVA followed by Bonferroni’s *post hoc* test and only significant rescue (*p* ≤ 0.05) of seizure behaviour is shown.

**Figure 4.**
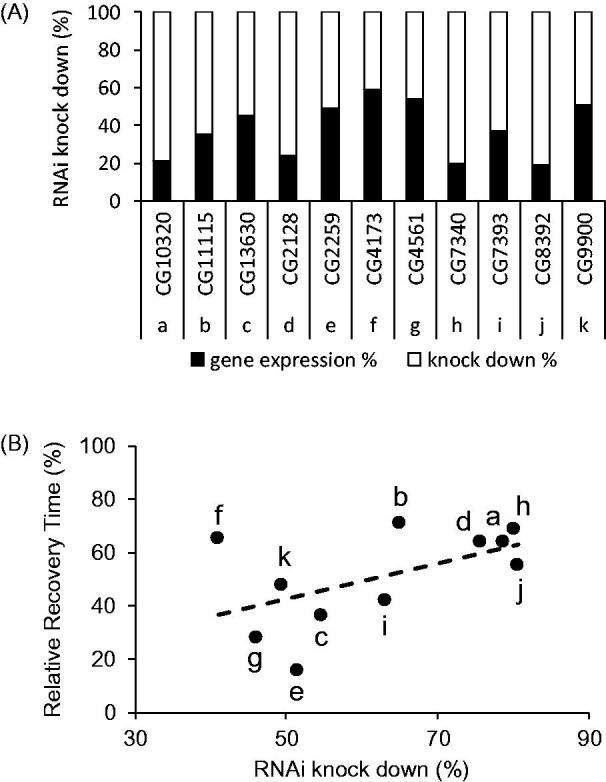
Efficacy of RNAi-mediated knockdown of gene expression does not correlate to seizure reduction in the *para^bss^* mutant. Male flies of 11 UAS-RNAi lines that spanned the effective range of seizure observed (cf. Figure 3) were crossed with *elav-*GAL4^C155^ females. The total RNA of F1 male fly heads (*elav-*GAL4^C155^/*Y*;UAS-RNAi/*+*) was extracted and quantitative RT-PCR performed to examine RNAi knockdown efficiency. (A) Black bars show gene expression percentage, while the complementary white bars show the RNAi knockdown percentage. RNAi knockdown efficiency ranges between 41 and 80%. The letters a-k and the corresponding CG numbers along the *x*-axis indicate the individual UAS-RNAi lines (Supplementary Table 4 for the detail of these genes). (B) RNAi knockdown efficacy plotted against relative recovery time (normalized to the controls *para^bss^*/Y;GAL4*^Cha(19B)^*/+, set at 100%) of each line tested in (A). The letters a-k indicate the corresponding CG numbers shown in (A). The line of best fit is not significantly different to a horizontal line (representing no correlation, Pearson’s correlation).

Increasing dPum expression and/or activity is anticonvulsant in *Drosophila* bs mutants (Lin *et al*., 2017). Thus, to verify that the 101 activity-dependent genes which, on knockdown, potentiate dPum activity have an anticonvulsant effect, we performed a behaviour screen in adult *para^bss^* flies. The *para^bss^* mutation is a missense allele (hypomorphic) of the sole *voltage-gated sodium channel* and exhibits robust seizure like behaviour when adult flies are exposed to strong sensory stimuli (e.g. vortexing) (Parker, Padilla, Du, Dong, & Tanouye, 2011). We expressed UAS-RNAi constructs in the *para^bss^* background, driving expression in cholinergic neurons (GAL4*^Cha(19B)^*) which is the principle excitatory neurotransmitter of the insect CNS. We determined the effectiveness of 94 RNAi candidates (available from Vienna *Drosophila* Resource Centre). Out of 94 RNAi lines expressed, 57 (∼61%) exhibited significant behavioural rescue of seizure duration (Figure 3 and see Supplementary Table 4 for full list). The different genetic backgrounds of the various RNAi lines may also influence seizure behavior. We did not first outcross these RNAi lines to generate similar genetic backgrounds, therefore, this influence is undetermined. To allow comparison of RNAi efficacy, we compared effect to that produced by phenytoin. Phenytoin is a potent AED (Keppel Hesselink, 2017) and shows good anticonvulsant effect in *Drosophila* (Lin *et al*., 2015). Thus, we also fed *para^bss^* adult flies with phenytoin (1.6 mM) for 24 h and tested seizure behaviour. Phenytoin fed flies exhibited significant reduction in seizure duration (64.2 ± 17.0%) compared to vehicle control (set at 100%, *n* = 5, *p* = .0049, *t*-test). Of the 57 RNAi lines tested, 42 exhibited similar or greater rescue effect compared to phenytoin (Figure 3). The most significant reduction was observed on knocking-down *Glutamate-cysteine ligase catalytic subunit* (*GCL*, the rate-limiting enzyme for glutathione synthesis (Franklin *et al*., 2009)). Similar significant rescues were observed for knock-down of *Bab Interacting Protein 1* (*bip1*) (Pointud, Larsson, Dastugue, & Couderc, 2001), *Tyrosyl-tRNA synthetase* or *Ribosomal protein S5a* (both core components of translational machinery (Schimmel and Soll, 1979)). To determine whether efficacy of seizure rescue is dictated by knockdown efficiency, we used quantitative RT-PCR to compare 11 randomly selected RNAi lines, driven by a pan-neuronal GAL4 line, *elav*-GAL4^C155^. Knockdown ranged between 41 to 80% (Figure 4(A)) but, importantly, did not significantly correlate to seizure reduction (the line fit is not significantly different from a ‘zero’ horizontal line, Pearson’s correlation) (Figure 4(B)). Thus, we conclude that anticonvulsive efficacy is dictated by targeted gene knock-down and not efficacy of individually-expressed RNAi’s .

### Rescue of seizure by chemical inhibitors

Our rationale to identify genes which, on knock-down, reduce seizure in *Drosophila* is that the same outcome should be recapitulated through pharmacological block of the gene-derived protein product. To test this, we identified known chemical inhibitors for a selection of genes identified and raised *para^bss^* mutant larvae on food containing these compounds (it is easier to feed drugs to larvae than to adults). Wall-climbing 3rd instar larvae were subjected to electric shock to test seizure severity (Marley and Baines, 2011). Although knock-down of *GCL*, *bip1*, *Tyrosyl-tRNA synthetase* or *Ribosomal protein S5a* showed the most effective seizure rescue effect (Figure 3), no chemical inhibitors that specifically inhibit these gene products are currently available. Therefore, we searched for chemical inhibitors which are well characterised and accessible. The drugs tested and their relevant targets are listed in Table 2. Exposure of *para^bss^* mutant larvae to phenytoin (1.6 mM) is sufficient, as expected, to produce a significant reduction in larval seizure duration (52.3 ± 15.1%) compared to vehicle control (set at 100%, *n* = 30, *p* = 1.4 × 10^−16^, *t*-test) mirroring the result of 24 h phenytoin feeding in adults (64.2 ± 17.0%). Mutant *para^bss^* larvae exposed to SB203580 or losmapimod (MAP kinase inhibitors), sodium fluoride (protein phosphatase inhibitor), gemcitabine (ribonucleoside diphosphate reductase inhibitor), metformin (inhibit NADH dehydrogenase activity), bestatin (aminopeptidase inhibitor), WP1066 (inhibitor of JAK-STAT signalling) or valproic acid (histone deacetylase inhibitor) similarly showed a significant reduction in seizure duration (ranging from 46 to 78%, set vehicle control at 100%) (Table 2). We also tested additional compounds that did not show significant anticonvulsive activity. These were: MG-132 (84 µM) (protease inhibitor), zaprinast (737 µM) (phosphodieasterase inhibitors), BEZ235 (213 µM) (inhibits PI3K and mTOR kinase activity), (±)-thalidomide (387 µM) (inhibits E3 ubiquitin ligase) or exthosuximide (1.4 mM) (T-type Ca^2+^ channel blocker). The concentrations stated were the maximum dose that larvae could tolerate. However, for all drugs tested, we are unable to determine the actual concentration of drug that reached the CNS. This is why we compare anticonvulsant effect achieved to phenytoin, which we use to standardize effect. The effectiveness of drugs, which target protein products of genes identified in our RNAi-screen, not only validates the screen methodology but, more importantly, identifies potential novel targets that may prove favourable for next-generation AEDs.

### Anticonvulsive effect is achieved through increased transcription of pumilio

**Figure 5.**
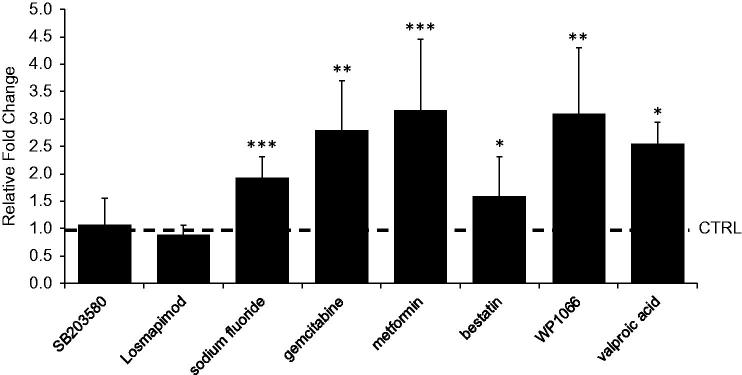
Identification of chemical inhibitors that enhanced transactivation of the *dpum* promoter. GAL4*^dpum^* was used to drive expression of UAS-*luciferase* (*luc*). Larvae grown on food containing sodium fluoride, gemcitabine, metformin, bestatin, WP1066 or valproic acid show significant increases in luc-activity (i.e. *dpum* promoter activity) compared to vehicle control (CTRL). Relative fold change, normalised to each vehicle control (set at 1), is shown. Values (*n* = 5, mean ± s.d.) were compared to each vehicle control by Student’s *t*-test and results were deemed significant at *p ≤ 0.05, **p ≤ 0.01 or ***p ≤ 0.001.

Our RNAi screen identified genes that, on knock-down, increase dPum activity. The mode of action for this effect may, conceivably, be increased expression of *dpum* (i.e. a transcriptional effect) or modification of protein function (i.e. post-translational modification). To begin to resolve this, we identified and cloned the *dpum* promoter region and placed it upstream of GAL4 (W.-H.L. and R.A.B., to be reported elsewhere). This was necessary because anti-Pum antibodies, whilst effective in mammals, do not work well in *Drosophila* (W.-H.L. and R.A.B., personal observations). Predictably, GAL4-mediated expression of GFP shows ubiquitous and low level pan-neuronal expression in 3rd instar CNS (W.-H.L. and R.A.B., unpublished observations). GAL4-mediated expression of luciferase allowed us to better quantitate expression levels. Raising transgenic larvae on food containing the proconvulsant PTX (1 µg/ml) was sufficient to result in a significant increase in luc-activity (1.7 ± 0.4-fold increase, compared to vehicle control, set at 1, *n* = 5, *p* = .003). This expected result validates that the *dpum* promoter is responsive to synaptic activity. Raising larvae on food containing sodium fluoride, gemcitabine, metformin, bestatin, WP1066 or valproic acid, respectively, resulted in 1.9 ± 0.4, 2.8 ± 0.9, 3.2 ± 1.3, 1.6 ± 0.7, 3.1 ± 1.2 and 2.5 ± 0.4-fold increase in luc-activity compared to vehicle control (set at 1) (*n* = 5, *p* = .001, .01, .0008, .02, .003 and .03, respectively) (Figure 5). However, we did not observe a notable change following exposure to MAPK inhibitors, SB203580 or losmapimod (1.1 ± 0.5 and 0.9 ± 0.2-fold, respectively, *n* = 5, *p* > .05). This result suggests that the anticonvulsant effect of sodium fluoride, gemcitabine, metformin and valproic acid is achieved, at least in part, through increased transcription of *dpum*.

## Discussion

The majority of AEDs target ion channels or neurotransmitter signalling to limit neuronal hyperexcitability. However, despite the availability of numerous drugs, ∼30% of epilepsy patients do not respond to drug treatment (Bradford, 1995; Loscher and Schmidt, 2011; Sillanpaa and Schmidt, 2006). Development of next-generation AEDs, that modify novel targets, is needed to meet this shortfall. Taking advantage of our previous demonstration that manipulating *dpum* expression effectively diminishes seizure behaviour in *Drosophila* (Lin *et al*., 2017), we conducted a genome-wide RNAi screen, using a luciferase-based reporter of dPum activity, and have identified 101 activity-dependent dPum regulators. Functional cluster analysis demonstrated that cellular response to DNA damage (36%) and regulation of cell cycle (36%) are the major pathways involved in regulation of dPum expression/activity. Notably, our previous FDA-approved drug library screen, which identified 12 compounds to enhance dPum activity, showed 5 compounds, cladribine, gemcitabine, floxuridine, clofarabine and bleomycin, that similarly influence DNA damage and/or DNA/RNA synthesis (Lin *et al*., 2017). Indeed, several ribosomal components, including Ribosomal protein S8 (RpS8), have been shown to associate with chromatin at active transcription sites and to associate with nascent transcripts to form ribonucleoprotein complexes that regulate transcript expression (Brogna, Sato, & Rosbash, 2002). RpS8 has also been identified in genomic-scale yeast two-hybrid analyses as a Bip1-interacting protein (Formstecher *et al*., 2005; Giot *et al*., 2003; Stark *et al*., 2006). In this study, we identified both Bip1 and RpS8 indicative that both may co-operate to regulate *dpum* expression. Knockdown of either *bip1* or *rps8* resulted in a dramatic reduction of seizure duration in *para^bss^* (reduced to 27% and 45%, respectively), suggesting a Bip1/RpS8 complex functions as a negative regulator of *dpum* transcription.

The results of this study, together with results from our previous work (Lin *et al*., 2017) identify a number of pathways that may prove favourable for the design of next-generation AEDs. These pathways include the following.

### Proteasomal protein catabolism

Epileptogenesis is associated with large-scale changes in protein expression which contribute to hyperexcitability-promoting alterations in neuronal networks and synaptic transmission (Pitkanen, Lukasiuk, Dudek, & Staley, 2015). The ubiquitin proteasome is one of the major proteolytic systems. Upregulation of proteasome subunits has been observed in animal seizure models and human epilepsy (Broekaart *et al*., 2017; Engel, Lucas, & Henshall, 2017; Engel *et al*., 2017; Lin *et al*., 2017). In our screen, knockdown of proteasome subunits (i.e. *alpha1*, *beta1*, *beta3*, *alpha5*, *beta6* and *beta7*) enhanced dPum activity suggesting that dPum is a proteasome target. Interestingly, rapamycin (an mTOR pathway inhibitor) treatment attenuated proteasome beta5 subunit expression in the electrical post-*status epilepticus* (SE) rat model, which was associated with a reduced seizure frequency (Broekaart *et al*., 2017). Rapamycin is an effective anticonvulsant in both flies and mammals. For example, acutely fed rapamycin reduces seizure duration in *bangsensitive* (a bs-mutant) adult flies (Lin *et al*., 2015), prevents the development of absence seizures in WAG/Rij rats (Russo *et al*., 2013), reduces kindling-induced seizure in Tsc1GFAPCKO mice (Zeng, Xu, Gutmann, & Wong, 2008), and kainite-induced *status epilepticus* in rats (Macias *et al*., 2013). Rapamycin is also an effective treatment for epilepsy in children suffering tuberous sclerosis (Canpolat *et al*., 2014). Taken together, these results imply that the anticonvulsant effect of rapamycin may be achieved, at least in part, by reducing proteasome activity that, in turn, may increase Pum activity.

### MAPK pathway

The mitogen-activated protein kinase (MAPK) family includes three pathways: the extracellular signal regulated kinase (ERK) pathway, the p38 pathway and the C-Jun N-terminal kinases (JNK) pathway. MAPK signalling has a significant role in epileptogenesis (Pernice, Schieweck, Kiebler, & Popper, 2016). For example, a transcriptomic analysis of brain tissue in human temporal lobe epilepsy (TLE) and mouse pilocarpine induced *status epilepticus* reported dysregulated gene expression involved in MAPK-signalling, including up-regulation of MAPK (Hansen, Sakamoto, Pelz, Impey, & Obrietan, 2014; Salman *et al*., 2017). Acute seizure induction, using kainic acid, leads to a rapid activation of ERK and p38 in mouse hippocampus. Pre-treatment with the ERK inhibitor PD98059 and p38 inhibitor SB203580 selectively reduces evoked seizures (Jiang *et al*., 2005). On the other hand, *p38α^+/−^* mice showed resistance to kainite-induced seizure (Namiki *et al*., 2007). These observations implicate that MAPK induction is critical for seizure generation. Our previous report identified the *p38b* transcript to be up-regulated in 3rd instar larvae exposed to PTX (Lin *et al*., 2017). In this study, we show that *p38b* knockdown enhances dPum activity and, in turn, reduces *para^bss^* seizure behaviour. The p38 MAPK inhibitors, SB203580 and losmapimod, are similarly sufficient to reduce seizure duration in *para^bss^*. However, these same inhibitors did not influence *dpum* promoter activity indicative that p38 MAPK may regulate dPum through an alternative mechanism (perhaps protein phosphorylation). Use of the NetPhos 3.1 server (http://www.cbs.dtu.dk/services/NetPhos/) (Blom, Gammeltoft, & Brunak, 1999; Blom, Sicheritz-Ponten, Gupta, Gammeltoft, & Brunak, 2004) identifies 13 different putative p38 MAPK phosphorylation sites in dPum (W.-H.L. and R.A.B., unpublished data).

### JAK/STAT pathway

We show that both knockdown of *CG4022* expression (an unknown gene) and ingestion of WP1066 (JAK-STAT inhibitor) effectively reduced seizure duration in *para^bss^*. These findings implicate JAK/STAT signalling to contribute to seizure, perhaps through regulation of *dpum* expression. CG4022 is predicted to be a component in the JAK/STAT signalling pathway (Muller, Kuttenkeuler, Gesellchen, Zeidler, & Boutros, 2005). JAK/STAT signalling transmits information from extracellular stimuli, often through interaction with receptor tyrosine kinase (RTK)/Ras/MAPK pathways, to the nucleus to affect gene expression (Rawlings, Rosler, & Harrison, 2004). The JAK/STAT pathway is up-regulated in both pilocarpine- and kainite-induced *status epilepticus* (Choi *et al*., 2003; Xu *et al*., 2011). Administration of the JAK/STAT inhibitor, WP1066, reduces the severity of pilocarpine-induced seizure and altered JAK/STAT downstream target transcript expression (Grabenstatter *et al*., 2014).

### Histone deacetylase

Epilepsy-induced alteration in gene expression is presumably guided by epigenetic mechanisms, including chromatin modification via DNA methylation and/or histone modification (Hwang, Aromolaran, & Zukin, 2013; Kobow and Blumcke, 2014; McClelland *et al*., 2011). Histone acetylation is catalysed by histone acetyltransferases and reversed by histone deacetylases (HDACs). In general, acetylated histones, H3 and H4, reflect a more permissive (open) state of chromatin allowing increased gene expression, whereas deacetylation mostly suppresses transcription (Kimura, Matsubara, & Horikoshi, 2005). Thus, HDAC inhibitors often serve to re-activate silenced genes (Butler and Kozikowski, 2008). We show that knockdown of *HDAC3* enhances both dPum activity and reduces seizure duration in *para^bss^*. This result implies that enhanced dPum activity, on *HDAC3* knockdown, may result from increased *dpum* transcription. On the other hand, rapid change in *HDAC* transcript expression has been demonstrated in both kainic acid- and pilocarpine-induced TLE mouse models (Jagirdar, Drexel, Bukovac, Tasan, & Sperk, 2015; Jagirdar, Drexel, Kirchmair, Tasan, & Sperk, 2015). These findings suggest *HDAC* expression responds to activity alteration and may thus be involved in epileptogenesis. Valproic acid inhibits HDAC activity (Gottlicher *et al*., 2001; Phiel *et al*., 2001) and is one of the most commonly used AEDs. However, the underlining mechanism of valproic acid remains uncertain. For example, valproic acid is reported to enhance GABA-receptor activation (Harrison and Simmonds, 1982), increase the synthesis of GABA by stimulating glutamate decarboxylase (GAD) (Nau and Loscher, 1982) and to modulate voltage-gated sodium channel steady-state inactivation kinetics (Vreugdenhil, van Veelen, van Rijen, Lopes da Silva, & Wadman, 1998). We show that valproic acid effectively ameliorates *para^bss^* 3rd instar larvae seizure duration and enhances *dpum* promoter activity. We postulate that the action of valproic acid, by inhibiting HDAC activity, increases *dpum* expression which, in turn, reduces neuron action potential firing. Intriguingly, valproic acid exposure decreases p300/CBP protein expression in mouse P19 cells (Lamparter and Winn, 2016). We also observed that knockdown of p300/CBP (*nejire*) is anticonvulsant.

## Conclusions

Epileptic seizures are associated with a pathological dysregulation of Pum expression. It has been shown that increasing *dpum* expression in *Drosophila* reduces seizure. We present a genome-wide RNAi screen that identifies 101 activity-dependent repressors of *dpum*. Expression of RNA interference (RNAi) *in vivo* shows that knockdown of 57 of these genes provides significant behavioural rescue of an induced-seizure phenotype in the *para^bss^* seizure mutant. We further show that chemical inhibitors, targeting the protein products of some of the identified genes, are similarly effective as anticonvulsants. Finally, we provide evidence to suggest that many of these chemical inhibitors act to enhance *dpum* expression.

## Supplementary Material

Supplemental Tables

## References

[CIT0001] Arvola R.M., Weidmann C.A., Tanaka Hall T.M., & Goldstrohm A.C. (2017). Combinatorial control of messenger RNAs by Pumilio, Nanos and brain tumor proteins. RNA Biology, 14, 1445–1456. doi:10.1080/15476286.2017.1306168 28318367 PMC5785226

[CIT0002] Baines R.A., Giachello C.N., & Lin W.H. (2017). Drosophila In Pitka¨nen A., Buckmaster P.S., Galanopoulou A. & Moshe´ S. (Eds.), Models of seizures and epilepsy (pp. 345–358). London: Elsevier/Academic Press.

[CIT0003] Bindea G., Galon J., & Mlecnik B. (2013). CluePedia Cytoscape plugin: pathway insights using integrated experimental and in silico data. Bioinformatics, 29, 661–663. doi:10.1093/bioinformatics/btt019 23325622 PMC3582273

[CIT0004] Bindea G., Mlecnik B., Hackl H., Charoentong P., Tosolini M., Kirilovsky A., … Galon J. (2009). ClueGO: a Cytoscape plug-in to decipher functionally grouped gene ontology and pathway annotation networks. Bioinformatics, 25, 1091–1093. doi:10.1093/bioinformatics/btp101 19237447 PMC2666812

[CIT0005] Blom N., Gammeltoft S., & Brunak S. (1999). Sequence and structure-based prediction of eukaryotic protein phosphorylation sites. Journal of Molecular Biology, 294, 1351–1362. doi:10.1006/jmbi.1999.3310 10600390

[CIT0006] Blom N., Sicheritz-Ponten T., Gupta R., Gammeltoft S., & Brunak S. (2004). Prediction of post-translational glycosylation and phosphorylation of proteins from the amino acid sequence. Proteomics, 4, 1633–1649. doi:10.1002/pmic.200300771 15174133

[CIT0007] Boutros M., Kiger A.A., Armknecht S., Kerr K., Hild M., Koch B., … Heidelberg Fly Array C. (2004). Genome-wide RNAi analysis of growth and viability in Drosophila cells. Science, 303, 832–835. doi:10.1126/science.1091266 14764878

[CIT0008] Bradford H.F. (1995). Glutamate, GABA and epilepsy. Progress in Neurobiology, 47, 477–511. doi:10.1016/0301-0082(95)00030-5 8787032

[CIT0009] Broekaart D.W.M., van Scheppingen J., Geijtenbeek K.W., Zuidberg M.R.J., Anink J.J., Baayen J.C., … van Vliet E.A. (2017). Increased expression of (immuno)proteasome subunits during epileptogenesis is attenuated by inhibition of the mammalian target of rapamycin pathway. Epilepsia, 58, 1462–1472. doi:10.1111/epi.13823 28643873

[CIT0010] Brogna S., Sato T.A., & Rosbash M. (2002). Ribosome components are associated with sites of transcription. Molecular Cell, 10, 93–104. doi:10.1016/S1097-2765(02)00565-8 12455503

[CIT0011] Butler K.V., & Kozikowski A.P. (2008). Chemical origins of isoform selectivity in histone deacetylase inhibitors. Current Pharmaceutical Design, 14, 505–528. Retrieved from https://www.ncbi.nlm.nih.gov/pubmed/18336297 10.2174/138161208783885353 18336297 10.2174/138161208783885353

[CIT0012] Canpolat M., Per H., Gumus H., Yikilmaz A., Unal E., Patiroglu T., … Kumandas S. (2014). Rapamycin has a beneficial effect on controlling epilepsy in children with tuberous sclerosis complex: results of 7 children from a cohort of 86. Childs Nervous System, 30, 227–240. doi:10.1007/s00381-013-2185-6 23743820

[CIT0013] Choi J.S., Kim S.Y., Park H.J., Cha J.H., Choi Y.S., Kang J.E., … Lee M.Y. (2003). Upregulation of gp130 and differential activation of STAT and p42/44 MAPK in the rat hippocampus following kainic acid-induced seizures. Brain Research Molecular Brain Research, 119, 10–18. doi:10.1016/j.molbrainres.2003.08.010 14597225

[CIT0014] Copmans D., Siekierska A., & de Witte P.A.M. (2017). Zebrafish models of epilepsy and epileptic seizures In Pitka¨nen A., Buckmaster P.S., Galanopoulou A. & Moshe´ S. (Eds.), Models of seizures and epilepsy (pp. 369–384). London: Elsevier/Academic Press.

[CIT0015] Driscoll H.E., Muraro N.I., He M., & Baines R.A. (2013). Pumilio-2 regulates translation of Nav1.6 to mediate homeostasis of membrane excitability. Journal of Neuroscience, 33, 9644–9654. doi:10.1523/JNEUROSCI.0921-13.2013 23739961 PMC3678506

[CIT0016] Engel T., Lucas J.J., & Henshall D.C. (2017). Targeting the proteasome in epilepsy. Oncotarget, 8, 45042–45043. doi:10.18632/oncotarget.18418 28611313 PMC5542164

[CIT0017] Engel T., Martinez-Villarreal J., Henke C., Jimenez-Mateos E.M., Sanz-Rodriguez A., Alves M., … Henshall D.C. (2017). Spatiotemporal progression of ubiquitin-proteasome system inhibition after status epilepticus suggests protective adaptation against hippocampal injury. Molecular Neurodegeneration, 12, 21. doi:10.1186/s13024-017-0163-2 28235423 PMC5324261

[CIT0018] Follwaczny P., Schieweck R., Riedemann T., Demleitner A., Straub T., Klemm A.H., … Kiebler M.A. (2017). Pumilio2-deficient mice show a predisposition for epilepsy. Disease Models and Mechanisms, 10, 1333–1342. doi:10.1242/dmm.029678 29046322 PMC5719250

[CIT0019] Formstecher E., Aresta S., Collura V., Hamburger A., Meil A., Trehin A., … Daviet L. (2005). Protein interaction mapping: a Drosophila case study. Genome Research, 15, 376–384. doi:10.1101/gr.2659105 15710747 PMC551564

[CIT0020] Franklin C.C., Backos D.S., Mohar I., White C.C., Forman H.J., & Kavanagh T.J. (2009). Structure, function, and post-translational regulation of the catalytic and modifier subunits of glutamate cysteine ligase. Molecular Aspects of Medicine, 30, 86–98. doi:10.1016/j.mam.2008.08.009 18812186 PMC2714364

[CIT0021] Giachello C.N., & Baines R.A. (2017). Regulation of motoneuron excitability and the setting of homeostatic limits. Current Opinion in Neurobiology, 43, 1–6. doi:10.1016/j.conb.2016.09.014 27721083

[CIT0022] Giot L., Bader J.S., Brouwer C., Chaudhuri A., Kuang B., Li Y., … Rothberg J.M. (2003). A protein interaction map of Drosophila melanogaster. Science, 302, 1727–1736. doi:10.1126/science.1090289 14605208

[CIT0023] Gottlicher M., Minucci S., Zhu P., Kramer O.H., Schimpf A., Giavara S., … Heinzel T. (2001). Valproic acid defines a novel class of HDAC inhibitors inducing differentiation of transformed cells. Embo Journal, 20, 6969–6978. doi:10.1093/emboj/20.24.6969 11742974 PMC125788

[CIT0024] Grabenstatter H.L., Del Angel Y.C., Carlsen J., Wempe M.F., White A.M., Cogswell M., … Brooks-Kayal A.R. (2014). The effect of STAT3 inhibition on status epilepticus and subsequent spontaneous seizures in the pilocarpine model of acquired epilepsy. Neurobiology of Disease, 62, 73–85. doi:10.1016/j.nbd.2013.09.003 24051278 PMC3908775

[CIT0025] Gupta Y.K., Lee T.H., Edwards T.A., Escalante C.R., Kadyrova L.Y., Wharton R.P., & Aggarwal A.K. (2009). Co-occupancy of two Pumilio molecules on a single hunchback NRE. RNA, 15, 1029–1035. doi:10.1261/rna.1327609 19372537 PMC2685510

[CIT0026] Hansen K.F., Sakamoto K., Pelz C., Impey S., & Obrietan K. (2014). Profiling status epilepticus-induced changes in hippocampal RNA expression using high-throughput RNA sequencing. Science Reports, 4, 6930. p doi:10.1038/srep06930 PMC4894418 25373493

[CIT0027] Harrison N.L., & Simmonds M.A. (1982). Sodium valproate enhances responses to GABA receptor activation only at high concentrations. Brain Research, 250, 201–204. doi:10.1016/0006-8993(82)90971-4 6291721

[CIT0028] Huang da W., Sherman B.T., & Lempicki R.A. (2009a). Bioinformatics enrichment tools: paths toward the comprehensive functional analysis of large gene lists. Nucleic Acids Research, 37, 1–13. doi:10.1093/nar/gkn923 19033363 PMC2615629

[CIT0029] Huang da W., Sherman B.T., & Lempicki R.A. (2009b). Systematic and integrative analysis of large gene lists using DAVID bioinformatics resources. Nature Protocols, 4, 44–57. doi:10.1038/nprot.2008.211 19131956

[CIT0030] Hwang J.Y., Aromolaran K.A., & Zukin R.S. (2013). Epigenetic mechanisms in stroke and epilepsy. Neuropsychopharmacology, 38, 167–182. doi:10.1038/npp.2012.134 22892394 PMC3521969

[CIT0031] Jagirdar R., Drexel M., Bukovac A., Tasan R.O., & Sperk G. (2015). Expression of class II HDACs in two mouse models of temporal lobe epilepsy. Journal of Neurochemistry, doi:10.1111/jnc.13440 PMC4738395 26603269

[CIT0032] Jagirdar R., Drexel M., Kirchmair E., Tasan R.O., & Sperk G. (2015). Rapid changes in expression of class I and IV histone deacetylases during epileptogenesis in mouse models of temporal lobe epilepsy. Experimetnal Neurology, 273, 92–104. doi:10.1016/j.expneurol.2015.07.026 26238735

[CIT0033] Jiang W., Van Cleemput J., Sheerin A.H., Ji S.P., Zhang Y., Saucier D.M., … Zhang X. (2005). Involvement of extracellular regulated kinase and p38 kinase in hippocampal seizure tolerance. Journal of Neuroscience Research, 81, 581–588. doi:10.1002/jnr.20566 15948190

[CIT0034] Keppel Hesselink J.M. (2017). Phenytoin: a step by step insight into its multiple mechanisms of action-80 years of mechanistic studies in neuropharmacology. Journal of Neurology, 264, 2043–2047. doi:10.1007/s00415-017-8465-4 28349209

[CIT0035] Kimura A., Matsubara K., & Horikoshi M. (2005). A decade of histone acetylation: marking eukaryotic chromosomes with specific codes. Journal of Biochemistry, 138, 647–662. doi:10.1093/jb/mvi184 16428293

[CIT0036] Kleino A., Valanne S., Ulvila J., Kallio J., Myllymaki H., Enwald H., … Ramet M. (2005). Inhibitor of apoptosis 2 and TAK1-binding protein are components of the Drosophila Imd pathway. Embo Journal, 24, 3423–3434. doi:10.1038/sj.emboj.7600807 16163390 PMC1276168

[CIT0037] Klitgaard H., Matagne A., Nicolas J.M., Gillard M., Lamberty Y., De Ryck M., … Kenda B. (2016). Brivaracetam: Rationale for discovery and preclinical profile of a selective SV2A ligand for epilepsy treatment. Epilepsia, 57, 538–548. doi:10.1111/epi.13340 26920914

[CIT0038] Kobow K., & Blumcke I. (2014). Epigenetic mechanisms in epilepsy. Progress in Brain Research, 213, 279–316. doi:10.1016/B978-0-444-63326-2.00014-4 25194494

[CIT0039] Lamparter C.L., & Winn L.M. (2016). Valproic acid exposure decreases Cbp/p300 protein expression and histone acetyltransferase activity in P19 cells. Toxicology and Applied Pharmacology, 306, 69–78. doi:10.1016/j.taap.2016.07.001 27381264

[CIT0040] Lason W., Chlebicka M., & Rejdak K. (2013). Research advances in basic mechanisms of seizures and antiepileptic drug action. Pharmacological Reports, 65, 787–801. doi:10.1016/S1734-1140(13)71060-0 24145073

[CIT0041] Lin W.H., & Baines R.A. (2015). Regulation of membrane excitability: a convergence on voltage-gated sodium conductance. Molecular Neurobiology, 51, 57–67. doi:10.1007/s12035-014-8674-0 24677068 PMC4309913

[CIT0042] Lin W.H., Giachello C.N., & Baines R.A. (2017). Seizure control through genetic and pharmacological manipulation of Pumilio in Drosophila: a key component of neuronal homeostasis. Disease Models and Mechanisms, 10, 141–150. doi:10.1242/dmm.027045 28067623 PMC5312004

[CIT0043] Lin W.H., He M., & Baines R.A. (2015). Seizure suppression through manipulating splicing of a voltage-gated sodium channel. Brain, 138, 891–901. doi:10.1093/brain/awv012 25681415 10.1093/brain/awv012 PMC5014079

[CIT0044] Loscher W., & Schmidt D. (2011). Modern antiepileptic drug development has failed to deliver: ways out of the current dilemma. Epilepsia, 52, 657–678. doi:10.1111/j.1528-1167.2011.03024.x 21426333

[CIT0045] Lykke K., Tollner K., Feit P.W., Erker T., MacAulay N., & Loscher W. (2016). The search for NKCC1-selective drugs for the treatment of epilepsy: Structure-function relationship of bumetanide and various bumetanide derivatives in inhibiting the human cation-chloride cotransporter NKCC1A. Epilepsy and Behavior, 59, 42–49. doi:10.1016/j.yebeh.2016.03.021 27088517

[CIT0046] Macias M., Blazejczyk M., Kazmierska P., Caban B., Skalecka A., Tarkowski B., … Jaworski J. (2013). Spatiotemporal characterization of mTOR kinase activity following kainic acid induced status epilepticus and analysis of rat brain response to chronic rapamycin treatment. PLoS One, 8, e64455. p doi:10.1371/journal.pone.0064455 23724051 PMC3665782

[CIT0047] Markstein M., Pitsouli C., Villalta C., Celniker S.E., & Perrimon N. (2008). Exploiting position effects and the gypsy retrovirus insulator to engineer precisely expressed transgenes. Nature Genetics, 40, 476–483. doi:10.1038/ng.101 18311141 PMC2330261

[CIT0048] Marley R., & Baines R.A. (2011). Increased persistent Na + current contributes to seizure in the slamdance bang-sensitive Drosophila mutant. Journal of Neurophysiology, 106, 18–29. doi:10.1152/jn.00808.2010 21451059 PMC3129721

[CIT0049] McClelland S., Flynn C., Dube C., Richichi C., Zha Q., Ghestem A., … Baram T.Z. (2011). Neuron-restrictive silencer factor-mediated hyperpolarization-activated cyclic nucleotide gated channelopathy in experimental temporal lobe epilepsy. Annals of Neurology, 70, 454–464. doi:10.1002/ana.22479 21905079 PMC3177145

[CIT0050] Mee C.J., Pym E.C., Moffat K.G., & Baines R.A. (2004). Regulation of neuronal excitability through pumilio-dependent control of a sodium channel gene. Journal of Neuroscience, 24, 8695–8703. doi:10.1523/JNEUROSCI.2282-04.2004 15470135 PMC6729971

[CIT0051] Muller P., Kuttenkeuler D., Gesellchen V., Zeidler M.P., & Boutros M. (2005). Identification of JAK/STAT signalling components by genome-wide RNA interference. Nature, 436, 871–875. doi:10.1038/nature03869 16094372

[CIT0052] Muraro N.I., Weston A.J., Gerber A.P., Luschnig S., Moffat K.G., & Baines R.A. (2008). Pumilio binds para mRNA and requires Nanos and Brat to regulate sodium current in Drosophila motoneurons. Journal of Neuroscience, 28, 2099–2109. doi:10.1523/JNEUROSCI.5092-07.2008 18305244 PMC2323674

[CIT0053] Namiki K., Nakamura A., Furuya M., Mizuhashi S., Matsuo Y., Tokuhara N., … Kasuya Y. (2007). Involvement of p38alpha in kainate-induced seizure and neuronal cell damage. Journal of Receptors and Signal Transduction Research, 27, 99–111. doi:10.1080/10799890701357855 17613723

[CIT0054] Nau H., & Loscher W. (1982). Valproic acid: brain and plasma levels of the drug and its metabolites, anticonvulsant effects and gamma-aminobutyric acid (GABA) metabolism in the mouse. Journal of Pharmacology and Experimental Therapeutics, 220, 654–659. Retrieved from https://www.ncbi.nlm.nih.gov/pubmed/6801254 6801254

[CIT0055] Noebels J. (2015). Pathway-driven discovery of epilepsy genes. Nature Neuroscience, 18, 344–350. doi:10.1038/nn.3933 25710836 PMC4852130

[CIT0056] Parker L., Padilla M., Du Y., Dong K., & Tanouye M.A. (2011). Drosophila as a model for epilepsy: bss is a gain-of-function mutation in the para sodium channel gene that leads to seizures. Genetics, 187, 523–534. doi:10.1534/genetics.110.123299 21115970 PMC3030494

[CIT0057] Pernice H.F., Schieweck R., Kiebler M.A., & Popper B. (2016). mTOR and MAPK: from localized translation control to epilepsy. BMC Neuroscience, 17, 73. p doi:10.1186/s12868-016-0308-1 27855659 PMC5114760

[CIT0058] Phiel C.J., Zhang F., Huang E.Y., Guenther M.G., Lazar M.A., & Klein P.S. (2001). Histone deacetylase is a direct target of valproic acid, a potent anticonvulsant, mood stabilizer, and teratogen. Journal of Biological Chemistry, 276, 36734–36741. doi:10.1074/jbc.M101287200 11473107

[CIT0059] Pitkanen A., Lukasiuk K., Dudek F.E., & Staley K.J. (2015). Epileptogenesis. Cold Spring Harbor Perspectives in Medicine, 5, a022822. doi:10.1101/cshperspect.a022822 26385090 PMC4588129

[CIT0060] Pointud J.C., Larsson J., Dastugue B., & Couderc J.L. (2001). The BTB/POZ domain of the regulatory proteins Bric a brac 1 (BAB1) and Bric a brac 2 (BAB2) interacts with the novel Drosophila TAF(II) factor BIP2/dTAF(II)155. Developmental Biology, 237, 368–380. doi:10.1006/dbio.2001.0358 11543621

[CIT0061] Ramet M., Manfruelli P., Pearson A., Mathey-Prevot B., & Ezekowitz R.A. (2002). Functional genomic analysis of phagocytosis and identification of a Drosophila receptor for E. coli. Nature, 416, 644–648. doi:10.1038/nature735 11912489

[CIT0062] Rawlings J.S., Rosler K.M., & Harrison D.A. (2004). The JAK/STAT signaling pathway. Journal of Cell Science, 117, 1281–1283. doi:10.1242/jcs.00963 15020666

[CIT0063] Russo E., Citraro R., Donato G., Camastra C., Iuliano R., Cuzzocrea S., … De Sarro G. (2013). mTOR inhibition modulates epileptogenesis, seizures and depressive behavior in a genetic rat model of absence epilepsy. Neuropharmacology, 69, 25–36. doi:10.1016/j.neuropharm.2012.09.019 23092918

[CIT0064] Saito R., Smoot M.E., Ono K., Ruscheinski J., Wang P.L., Lotia S., … Ideker T. (2012). A travel guide to Cytoscape plugins. Nature Methods, 9, 1069–1076. doi:10.1038/nmeth.2212 23132118 PMC3649846

[CIT0065] Salman M.M., Sheilabi M.A., Bhattacharyya D., Kitchen P., Conner A.C., Bill R.M., … Princivalle A.P. (2017). Transcriptome analysis suggests a role for the differential expression of cerebral aquaporins and the MAPK signalling pathway in human temporal lobe epilepsy. European Journal of Neuroscience, 46, 2121–2132. doi:10.1111/ejn.13652 28715131

[CIT0066] Schimmel P.R., & Soll D. (1979). Aminoacyl-tRNA synthetases: general features and recognition of transfer RNAs. Annual Review of Biochemistry, 48, 601–648. doi:10.1146/annurev.bi.48.070179.003125 382994

[CIT0067] Schneider I. (1972). Cell lines derived from late embryonic stages of Drosophila melanogaster. Journal of Embryology and Experimental Morphology, 27, 353–365. Retrieved from https://www.ncbi.nlm.nih.gov/pubmed/4625067 4625067

[CIT0068] Shannon P., Markiel A., Ozier O., Baliga N.S., Wang J.T., Ramage D., … Ideker T. (2003). Cytoscape: a software environment for integrated models of biomolecular interaction networks. Genome Research, 13, 2498–2504. doi:10.1101/gr.1239303 14597658 PMC403769

[CIT0069] Siemen H., Colas D., Heller H.C., Brustle O., & Pera R.A. (2011). Pumilio-2 function in the mouse nervous system. PLoS One, 6, e25932. doi:10.1371/journal.pone.0025932 22016787 PMC3189250

[CIT0070] Sillanpaa M., & Schmidt D. (2006). Natural history of treated childhood-onset epilepsy: prospective, long-term population-based study. Brain, 129, 617–624. doi:10.1093/brain/awh726 16401617

[CIT0071] Stark C., Breitkreutz B.J., Reguly T., Boucher L., Breitkreutz A., & Tyers M. (2006). BioGRID: a general repository for interaction datasets. Nucleic Acids Research, 34, D535–D539. doi:10.1093/nar/gkj109 16381927 PMC1347471

[CIT0072] Takayanagi-Kiya S., & Jin Y. (2017). Nematode C. elegans: genetic dissection of pathways regulating seizure and epileptic-like behaviors In Pitka¨nen A., Buckmaster P.S., Galanopoulou A. & Moshe´ S. (Eds.), Models of seizures and epilepsy (pp. 327–344). London: Elsevier/Academic Press.

[CIT0073] Turrigiano G.G., & Nelson S.B. (1998). Thinking globally, acting locally: AMPA receptor turnover and synaptic strength. Neuron, 21, 933–935. Retrieved from https://www.ncbi.nlm.nih.gov/pubmed/9856445 10.1016/S0896-6273(00)80607-8 9856445 10.1016/s0896-6273(00)80607-8

[CIT0074] Vessey J.P., Vaccani A., Xie Y., Dahm R., Karra D., Kiebler M.A., & Macchi P. (2006). Dendritic localization of the translational repressor Pumilio 2 and its contribution to dendritic stress granules. Journal of Neuroscience, 26, 6496–6508. doi:10.1523/JNEUROSCI.0649-06.2006 16775137 PMC6674044

[CIT0075] Vreugdenhil M., van Veelen C.W., van Rijen P.C., Lopes da Silva F.H., & Wadman W.J. (1998). Effect of valproic acid on sodium currents in cortical neurons from patients with pharmaco-resistant temporal lobe epilepsy. Epilepsy Research, 32, 309–320. Retrieved from https://www.ncbi.nlm.nih.gov/pubmed/9761330 10.1016/S0920-1211(98)00061-8 9761330 10.1016/s0920-1211(98)00061-8

[CIT0076] Wharton R.P., Sonoda J., Lee T., Patterson M., & Murata Y. (1998). The Pumilio RNA-binding domain is also a translational regulator. Molecular Cell, 1, 863–872. Retrieved from https://www.ncbi.nlm.nih.gov/pubmed/9660969 10.1016/S1097-2765(00)80085-4 9660969 10.1016/s1097-2765(00)80085-4

[CIT0077] Wickens M., Bernstein D.S., Kimble J., & Parker R. (2002). A PUF family portrait: 3'UTR regulation as a way of life. Trends in Genetics, 18, 150–157. Retrieved from https://www.ncbi.nlm.nih.gov/pubmed/11858839 11858839 10.1016/s0168-9525(01)02616-6

[CIT0078] Wreden C., Verrotti A.C., Schisa J.A., Lieberfarb M.E., & Strickland S. (1997). Nanos and pumilio establish embryonic polarity in Drosophila by promoting posterior deadenylation of hunchback mRNA. Development, 124, 3015–3023. Retrieved from https://www.ncbi.nlm.nih.gov/pubmed/9247343 9247343 10.1242/dev.124.15.3015

[CIT0079] Wu X.L., Huang H., Huang Y.Y., Yuan J.X., Zhou X., & Chen Y.M. (2015). Reduced Pumilio-2 expression in patients with temporal lobe epilepsy and in the lithium-pilocarpine induced epilepsy rat model. Epilepsy Behavior, 50, 31–39. doi:10.1016/j.yebeh.2015.05.017 26101106

[CIT0080] Xu Z., Xue T., Zhang Z., Wang X., Xu P., Zhang J., … Chen Y. (2011). Role of signal transducer and activator of transcription-3 in up-regulation of GFAP after epilepsy. Neurochemical Research, 36, 2208–2215. doi:10.1007/s11064-011-0576-1 21833841

[CIT0081] Yanagawa S., Lee J.S., & Ishimoto A. (1998). Identification and characterization of a novel line of Drosophila Schneider S2 cells that respond to wingless signaling. The Journal of Biological Chemistry, 273, 32353–32359. Retrieved from https://www.ncbi.nlm.nih.gov/pubmed/9822716 10.1074/jbc.273.48.32353 9822716 10.1074/jbc.273.48.32353

[CIT0082] Zamore P.D., Williamson J.R., & Lehmann R. (1997). The Pumilio protein binds RNA through a conserved domain that defines a new class of RNA-binding proteins. RNA, 3, 1421–1433. Retrieved from https://www.ncbi.nlm.nih.gov/pubmed/9404893 9404893 PMC1369583

[CIT0083] Zeng L.H., Xu L., Gutmann D.H., & Wong M. (2008). Rapamycin prevents epilepsy in a mouse model of tuberous sclerosis complex. Annals of Neurology, 63, 444–453. doi:10.1002/ana.21331 18389497 PMC3937593

