# Supplementary material for: An RNAi-mediated screen identifies novel targets for next-generation antiepileptic drugs based on increased expression of the homeostatic regulator pumilio
Source: J Neurogenet. 2018 May 2;32(2):106–17. doi: 10.1080/01677063.2018.1465570 (PMC5989157; doi:10.1080/01677063.2018.1465570)
Supplement: Supplemental Tables [file INEG_A_1465570_SM8434.zip › STable_2.pdf]

STable 2. *dpum* regulators

716 dsRNAs (699 genes) that increase dPum activity

BKN#	CG#	Flybase_ID	gene name	human orthologs	FF-PRE/Ren ratio
BKN26631	CG9786	FBgn0001180	hunchback	IKZF4, ZNF827, ZNF513, REST, IKZF5, ZI	0.04
BKN20079	CG6222	FBgn0003575	suppressor of sable	CPSF4L, ZC3H6, ZC3H8, CPSF4, ZC3H4	0.12
BKN31766	CG33516	FBgn0053516	defective proboscis extension response 3	KIRREL2, IGSF9B, KIRREL3, LRIT3, USH	0.13
BKN45849	CG5726	FBgn0034313	-	-	0.13
BKN29923	CG33526	FBgn0053526	PNUTS	PRR3, PPP1R10	0.13
BKN29609	CG12249	FBgn0021776	miranda	SLMAP	0.13
BKN31742	CG9983	FBgn0001215	Heterogeneous nuclear ribonucleoprotein at 98DE	DAZAP1, SLIRP, HNRNPD, HNRNPA1L2,	0.13
BKN29705	CG7843	FBgn0033062	Arsenic resistance protein 2	SRRT	0.16
BKN21707	CG9753	FBgn0039747	Adenosine receptor	ADORA1, GPBAR1, ADORA2A, ADORA2I	0.17
BKN21880	CG32435	FBgn0021760	chromosome bows	CLASP1, CLASP2	0.18
BKN20625	CG33106	FBgn0043884	multiple ankyrin repeats single KH domain	ANKRD17, ANKRD22, ANKHD1-EIF4EBP	0.19
BKN28162	CG12749	FBgn0004237	Heterogeneous nuclear ribonucleoprotein at 87F	MSI1, HNRNPA1L2, DAZAP1, SRSF10, B	0.20
BKN29801	CG13941	FBgn0033928	Arc2	OARD1, ARC	0.21
BKN20175	CG7340	FBgn0040493	granny smith	LAP3, NPEPL1	0.21
BKN27983	CG15218	FBgn0025674	Cyclin K	CCNT1, FAM58A, CCNK, CCNT2	0.21
BKN21793	CG7597	FBgn0037093	Cyclin-dependent kinase 12	CDK9, CDK12, CDK13	0.22
BKN20274	CG5520	FBgn0039562	Glycoprotein 93	HSP90AB1, HSP90AA1, HSP90B1	0.22
BKN28019	CG1770	FBgn0041210	Histone deacetylase 4	HDAC10, HDAC5, HDAC7, HDAC9, HDAC	0.22
BKN31529	CG12767	FBgn0040465	Dorsal interacting protein 3	-	0.23
BKN26940	CG14543	FBgn0039404	-	TSR2	0.23
BKN29305	CG10674	FBgn0035592	-	WDR83OS	0.24
BKN23854	CG32816	FBgn0052816	-	-	0.24
BKN21601	CG3497	FBgn0004837	Suppressor of Hairless	RBPJ, RBPJL	0.24
BKN45044	CG10281	FBgn0010282	Transcription factor IIFalpha	GTF2F1	0.26
BKN31453	CG7423	FBgn0030982	-	ANKRD7, ASB12, LUZP6, MTPN	0.26
BKN45759	CG6538	FBgn0010421	Transcription factor TFIIFbeta	GTF2F2	0.26
BKN21840	CG10907	FBgn0036207	-	CWC27	0.26
BKN23604	CG7347	FBgn0002901	mutagen-sensitive 304	ATRIP	0.27
BKN45175	CG2669	FBgn0086695	humpty dumpty	DONSON, ATP5O	0.27
BKN21346	CG17293	FBgn0032030	WD repeat domain 82	TWF2, TLR9, TWF1, WDR82	0.27
BKN30230	CG2129	FBgn0030008	-	ZNF775, ZNF276, ZBTB43, ZNF653, ZSC	0.28
BKN20728	CG2253	FBgn0029992	Upf2	UPF2	0.28
BKN20738	CG7082	FBgn0031401	papi	TDRD15, TDRD1, TDRKH, AKAP1, TDRD	0.29
BKN29127	CG11246	FBgn0037121	Rpb8	POLR2H	0.30
BKN27394	CG9170	FBgn0030716	-	CEP164	0.31
BKN23549	CG3893	FBgn0036826	asterix	GTSF1L, GTSF1	0.31
BKN24073	CG32375	FBgn0085445	Ankyrin 2	ANKRD65, TNKS, ANK3, ANKRD60, ANK	0.31
BKN46476	CG5193	FBgn0004915	Transcription factor IIB	GTF2B	0.31
BKN20359	CG11979	FBgn0033571	Rpb5	POLR2E	0.32
BKN46237	CG4746	FBgn0029003	mab-21	MAB21L1, MAB21L3, MB21D1, C2orf54, T	0.32
BKN20068	CG9750	FBgn0040075	reptin	RUVBL2, RUVBL1, SCCPDH	0.32
BKN20892	CG7035	FBgn0022942	cap binding protein 80	NCBP1	0.32
BKN29729	CG1397	FBgn0030275	retroactive	-	0.32
BKN29051	CG3279	FBgn0035156	Vesicle transport through interaction with t-SNAREs 1	VTI1B, VTI1A	0.33
BKN45546	CG31390	FBgn0051390	Mediator complex subunit 7	MED7	0.33
BKN27339	CG31390	FBgn0051390	Mediator complex subunit 7	MED7	0.33
BKN28067	CG8611	FBgn0027602	-	DDX31	0.33
BKN21235	CG1793	FBgn0039923	Mediator complex subunit 26	TCEA3, TCEA2, TCEA1, TCEANC, MED2	0.33
BKN27645	CG6647	FBgn0004363	porin	VDAC1, VDAC2, VDAC3	0.33
BKN31280	CG17763	FBgn0250904	-	MYL1, MYL6, MYL6B, MYL4, MYL3	0.34
BKN28886	CG10601	FBgn0014343	mirror	TGIF2-C20orf24, MKX, MEIS2, TGIF2LX, I	0.34
BKN25495	CG3814	FBgn0025692	Lifeguard	FAIM2, TMBIM1, TMBIM4, GRINA	0.34
BKN27045	CG31757	FBgn0259225	Phosphodiesterase 1c	PDE1A, PDE1B, PDE1C	0.34
BKN27827	CG13867	FBgn0034503	Mediator complex subunit 8	MED8	0.34
BKN20996	CG4976	FBgn0039559	Nuclear receptor binding SET domain protein	KMT2C, NSD1, SETD1B, ASH1L, PHF20,	0.34
BKN28331	CG6391	FBgn0036111	Aps	NUDT11, NUDT3, NUDT4, NUDT10	0.34

BKN20743	CG6761	FBgn0036031	-	KIAA1841	0.34
BKN29448	CG12357	FBgn0022943	cap binding protein 20	NCBP2	0.34
BKN27357	CG6948	FBgn0024814	Clathrin light chain	CLTB, CLTA	0.34
BKN42698	CR33736	FBgn0053736	snmRNA:430:CR33736	-	0.35
BKN24095	CG1433	FBgn0019637	Another transcription unit	LEO1	0.35
BKN22945	CG1245	FBgn0037359	Mediator complex subunit 27	MED27	0.35
BKN29118	CG13628	FBgn0039218	Rpb10	POLR2L	0.35
BKN22183	CG8954	FBgn0019890	Smg5	SMG5, SMG7	0.35
BKN22349	CG5193	FBgn0004915	Transcription factor IIB	GTF2B	0.35
BKN22010	CG9473	FBgn0024330	Mediator complex subunit 6	MED6	0.35
BKN29078	CG11184	FBgn0034923	Upf3	UPF3B, UPF3A	0.35
BKN40707	CG17397	FBgn0040020	Mediator complex subunit 21	MED21	0.35
BKN29031	CG5543	FBgn0034908	-	WDR70	0.36
BKN22748	CG3710	FBgn0010422	RNA polymerase II elongation factor	MED26, TCEANC2, TCEA3, TCEA2, TCE/	0.36
BKN25376	CG7172	FBgn0037102	CR6-interacting factor	GADD45GIP1	0.36
BKN26823	CG1421	FBgn0032966	-	-	0.36
BKN28041	CG11981	FBgn0026380	Proteasome beta3 subunit	PSMB3	0.37
BKN20511	CG8487	FBgn0033714	gartenzwerg	GBF1, NCF4	0.37
BKN41369	CG45782	FBgn0069947	-	SLC45A4, SLC45A2, SLC45A1, SLC45A3	0.37
BKN21393	CG9431	FBgn0032484	kekkon4	ASPN, TPBG, LRRC26, SLITRK5, OMD, A	0.37
BKN21474	CG5923	FBgn0005696	DNA polymerase alpha 73kD	POLA2	0.37
BKN23064	CG9494	FBgn0032074	Tetraspanin 29Fa	TSPAN7, CD151, TSPAN6, CD63, TSPAN	0.37
BKN31525	CG13055	FBgn0036583	-	-	0.37
BKN28341	CG32579	FBgn0052579	-	XKR8, XKR5, XKR6, XKR7, XKR4, XKR9	0.38
BKN21199	CG4003	FBgn0040078	pontin	RUVBL2, SCCPDH, RUVBL1	0.38
BKN33278	CG9163	FBgn0259110	mind-meld	ADAM8, ADAM19, ADAMDEC1, ADAM11,	0.38
BKN20442	CG8610	FBgn0012058	Cell division cycle 27	CDC27, TTC37	0.38
BKN24191	CG6071	FBgn0036186	-	ANPEP, ERAP1, ENPEP, ERAP2, TRHDE	0.38
BKN27744	CG5057	FBgn0036581	Mediator complex subunit 10	MED10	0.38
BKN27400	CG15010	FBgn0041171	archipelago	DAW1, BTRC, TRAF7, FBXW11, FBXW7	0.38
BKN46549	CG5057	FBgn0036581	Mediator complex subunit 10	MED10	0.38
BKN33859	HDC03660		-	-	0.39
BKN27828	CG1559	FBgn0030354	Upf1	ZGRF1, UPF1	0.39
BKN45246	CG18398	FBgn0032728	Transport and Golgi organization 6	TANGO6	0.39
BKN29165	CG15929	FBgn0029800	lin-52	LIN52	0.39
BKN30897	CG17575	FBgn0250842	-	CRISP1, GLIPR2, CRISPLD2, PI16, CLEC	0.39
BKN26681	CG18398	FBgn0032728	Transport and Golgi organization 6	TANGO6	0.40
BKN41948	CG34186	FBgn0085215	Rpb12	POLR2K	0.40
BKN20763	CG11115	FBgn0037202	Suppressor of stem-loop mutation	GTF2H2C_2, GTF2H2C, GTF2H2	0.40
BKN27092	CG11125	FBgn0033174	-	ENKD1	0.40
BKN41517	CG11430	FBgn0041585	olf186-F	ORAI2, ORAI3, ORAI1	0.40
BKN27693	CG7885	FBgn0026373	RNA polymerase II 33kD subunit	POLR2C	0.40
BKN46590	CG15155	FBgn0032669	-	GLYATL3	0.40
BKN27914	CG31155	FBgn0051155	Rpb7	POLR2G	0.40
BKN27453	CG1163	FBgn0003275	RNA polymerase II 18kD subunit	POLR2F	0.41
BKN22798	CG12031	FBgn0035145	Mediator complex subunit 14	MED14	0.41
BKN28221	CG1276	FBgn0015829	Transcription factor IIEbeta	GTF2E2	0.41
BKN28214	CG10938	FBgn0016697	Proteasome alpha5 subunit	PSMA8, PSMA5, PSMA2, PSMA7	0.41
BKN30058	CG17932	FBgn0040260	Ugt36Bc	UGT3A2, UGT2A3, UGT1A8, UGT1A3, UC	0.41
BKN20479	CG6369	FBgn0039260	Smg6	SMG6, SWT1	0.41
BKN42043	CG18816	FBgn0042086	Tetraspanin 42Eb	CD63	0.42
BKN21171	CG11990	FBgn0037657	hyrax	CDC73	0.42
BKN20571	CG6331	FBgn0019952	Organic cation transporter	SLC22A7, SLC22A13, SLC22A8, SLC22A	0.42
BKN27263	CG14714	FBgn0259227	-	PTPRC, PTPN23, PTPN2, PTPN7, PTPRC	0.42
BKN33257	CG14340	FBgn0031302	-	RGS9BP, RGS7BP	0.42
BKN23028	CG6325	FBgn0037814	-	DCAF15	0.42
BKN23929	CG3773	FBgn0038692	-	GCOM1, POLR2M	0.42
BKN31369	CG33520	FBgn0053520	transcriptional Adaptor 2a	TADA2A, POLR2D, TADA2B	0.42
BKN21812	CG14043	FBgn0031659	-	KIF1BP	0.42
BKN28878	CG6066	FBgn0039488	-	NKAP, NKAPL	0.42
BKN32668	CG33335	FBgn0053335	-	-	0.42
BKN20467	CG9518	FBgn0030590	-	CHDH	0.43

BKN41161	CG33667	FBgn0053667	-	-	0.43
BKN41361	CR32272	FBgn0052272	transfer RNA:Lysine-CTT 1-13	-	0.43
BKN24046	CG7031	FBgn0039027	-	GGN, TPRX1	0.43
BKN41466	CG33998	FBgn0053998	-	-	0.43
BKN20457	CG8625	FBgn0011604	Imitation SWI	SMARCA1, SMARCA5, CHD1L	0.43
BKN20417	CG4067	FBgn0020385	pugilist	MTHFD2L, MTHFD2, MTHFD1, MTHFD1L	0.43
BKN20166	CG9383	FBgn0029094	anti-silencing factor 1	ASF1A, ASF1B	0.44
BKN41323	CG33829	FBgn0053829	His2A:CG33829	H2AFJ, H2AFY2, HIST2H2AA3, HIST1H2/	0.44
BKN40320	CG30382	FBgn0050382	-	PSMA6	0.44
BKN20659	CG4152	FBgn0001986	Mtr4 helicase	SKIV2L2, SKIV2L, DDX60L	0.44
BKN30375	CG7592	FBgn0039685	Odorant-binding protein 99b	-	0.44
BKN45004	CG2684	FBgn0002542	lodestar	TTF2, HLTF	0.44
BKN24933	CG1738	FBgn0030291	-	C1orf131	0.44
BKN23026	CG4236	FBgn0015610	Chromatin assembly factor 1, p55 subunit	WDR59, WDR73, RBBP7, RBBP4	0.44
BKN20755	CG1250	FBgn0037357	Secretory 23	SEC23B, SEC23A	0.44
BKN22958	CG3735	FBgn0034933	-	DIEXF	0.44
BKN21334	CG8151	FBgn0033929	Transcription factor B1	GTF2H1	0.44
BKN42716	CR33742	FBgn0053742	snmRNA:430:CR33742	-	0.44
BKN34310	HDC17124		-	-	0.44
BKN20461	CG32743	FBgn0052743	no-on-and-no-off transient C	BOLA2-SMG1P6, BOLA2B, BOLA2, PRKD	0.44
BKN22002	CG10582	FBgn0028402	Sex-lethal interactor	LOC101060521, POLR3E	0.44
BKN22538	CG3376	FBgn0034997	-	SMPDL3B, SMPDL3A, SMPD1	0.45
BKN22413	CG12173	FBgn0037305	-	ENOPH1	0.45
BKN41731	CG41433	FBgn0084000	-	-	0.45
BKN41259	CG34235	FBgn0085264	-	-	0.45
BKN24577	CG4824	FBgn0000182	Bicaudal C	ANKS6, HDLBP, BICC1	0.45
BKN30372	CG14627	FBgn0040361	-	-	0.45
BKN23794	CG17063	FBgn0027107	Innexin 6	-	0.45
BKN22670	CG8392	FBgn0010590	Proteasome beta1 subunit	PSMB9, PSMB6	0.45
BKN21140	CG5790	FBgn0032677	-	CDC7	0.45
BKN41830	CG12029	FBgn0035454	dendritic arbor reduction 1	KLF1, KLF16, KLF2, SP5, KLF10, SP1, ZN	0.45
BKN27592	CG8445	FBgn0034088	calypso	BAP1, UCHL5	0.45
BKN46221	CG11981	FBgn0026380	Proteasome beta3 subunit	PSMB3	0.45
BKN45327	CG17917	FBgn0037431	-	PEBP1, MRPL38, PEBP4	0.46
BKN23071	CG14366	FBgn0085412	-	CNKSR1, PPP1R9A, PPP1R9B, PLEKHA4	0.46
BKN22426	CG4351	FBgn0030448	-	CHPF2, CHPF	0.46
BKN21295	CG31871	FBgn0051871	-	LIPK, LIPA, LIPN, LIPJ, LIPM, LIPF	0.46
BKN31003	CG11105	FBgn0030281	-	RCVRN, KCNIP1, USP32, KCNIP3, KCNIF	0.46
BKN42723	CR33745	FBgn0053745	snmRNA:430:CR33745	-	0.46
BKN46436	CG16753	FBgn0035393	-	FAM207A	0.46
BKN24170	CG30049	FBgn0050049	-	ERMP1	0.46
BKN22409	CG10682	FBgn0027936	vihar	UBE2U, UBE2C	0.46
BKN21396	CG13626	FBgn0039212	Syntaxin 18	STX18	0.46
BKN27575	CG12212	FBgn0003053	pebbled	ZNF217, ZNF821, ZNF536, RREB1, ZBTB	0.46
BKN41636	CG2048	FBgn0002413	discs overgrown	VRK1, TTBK1, CSNK1E, VRK2, CSNK1D,	0.47
BKN29858	CG11145	FBgn0033168	-	-	0.47
BKN22509	CG10565	FBgn0037051	-	DNAJB4, DNAJC2	0.47
BKN28462	CG13316	FBgn0023215	Mnt	MNT, MXI1, MXD3	0.47
BKN27924	CG6474	FBgn0000617	enhancer of yellow 1	TAF9, TAF9B	0.47
BKN22317	CG30359	FBgn0050359	Maltase A5	SLC3A1, SLC3A2	0.47
BKN27287	CG17999	FBgn0034552	-	ACSF3, ACSBG1, AACS	0.47
BKN23035	CG5514	FBgn0039560	Biorientation Defective 1	BOD1, BOD1L2, BOD1L1	0.47
BKN31868	CG8881	FBgn0026176	SKP1-related B	SKP1	0.47
BKN31307	CG4843	FBgn0004117	Tropomyosin 2	TPM3, TPM4, TPM1, TPM2	0.47
BKN45942	CG11184	FBgn0034923	Upf3	UPF3B, UPF3A	0.47
BKN46429	CR31845	FBgn0051845	-	-	0.47
BKN46472	CG3710	FBgn0010422	RNA polymerase II elongation factor	MED26, TCEANC2, TCEA3, TCEA2, TCE/	0.47
BKN29138	CG6840	FBgn0032634	Rpb11	POLR2J2, POLR2J, POLR2J3	0.47
BKN27226	CG31829	FBgn0051829	Cullin 3	CUL4A, CUL3, CACUL1, CUL4B	0.47
BKN28126	CG8156	FBgn0013750	ADP ribosylation factor at 51F	ARL11, ARF6, ARL15	0.48
BKN40065	CG40844	FBgn0085524	-	-	0.48
BKN21933	CG8912	FBgn0014870	P-element somatic inhibitor	FUBP1, FUBP3, KHSRP	0.48

BKN20492	CG1886	FBgn0030343	ATP7	ATP7B, ATP7A	0.48
BKN23761	CG3515	FBgn0031431	-	WDR82	0.48
BKN27040	CG12763	FBgn0004240	Diptericin A	-	0.48
BKN30760	CG10488	FBgn0000625	eyegone	PAX3, DUX4, PAX6, PAX7, DUXA, MIXL1,	0.48
BKN46390	CG6884	FBgn0036811	Mediator complex subunit 11	MED11	0.48
BKN20586	CG16903	FBgn0040394	-	CCNL2, CCNL1	0.48
BKN28922	CG6593	FBgn0003134	Protein phosphatase 1alpha at 96A	PPP1CB, PPP1CA, PPP1CC	0.48
BKN26226	CG9797	FBgn0037621	Motif 1 Binding Protein	ZNF98, ZNF485, ZNF784, ZNF28, ZNF850	0.48
BKN29435	CG6814	FBgn0020407	asunder	ASUN	0.48
BKN28385	CG10315	FBgn0034858	eukaryotic translation initiation factor 2B subunit delta	EIF2B4	0.48
BKN46538	CG10523	FBgn0041100	parkin	PARK2	0.48
BKN26385	CG14712	FBgn0037924	-	POM121L2, POM121, NUP153, POM121C	0.48
BKN26720	CG3363	FBgn0034987	over compensating males	TBX10, TBX1	0.48
BKN22908	CG4785	FBgn0031314	Integrator 14	DNAJC2, VWA9	0.48
BKN28002	CG9327	FBgn0003150	Proteasome alpha3 subunit	PSMB3, PSMA4	0.48
BKN27100	CG1464	FBgn0005558	eyeless	PITX3, PAX6, SHOX, LPXN, PDLIM3, HOX	0.48
BKN21658	CG2259	FBgn0040319	Glutamate-cysteine ligase catalytic subunit	GCLC	0.48
BKN32455	CG13845	FBgn0085405	-	NACC1, NACC2, ZBTB9	0.48
BKN25009	CG14352	FBgn0031351	-	-	0.49
BKN28276	CG10415	FBgn0015828	Transcription factor IIEalpha	GTF2E1	0.49
BKN40299	CG18495	FBgn0026781	Proteasome alpha1 subunit	PSMA6	0.49
BKN41307	CG33817	FBgn0053817	His2A:CG33817	HIST1H2AK, H2AFB3, H2AFB1, HIST2H2A	0.49
BKN28068	CG7564	FBgn0036734	-	LUC7L2, LUC7L3, LUC7L	0.49
BKN21317	CG18780	FBgn0013531	Mediator complex subunit 20	MED20	0.49
BKN22076	CG32654	FBgn0052654	Secretory 16	SEC16B, SEC16A	0.49
BKN28881	CG4817	FBgn0010278	Structure specific recognition protein	SSRP1	0.49
BKN28018	CG14981	FBgn0035473	maggie	TOMM22	0.49
BKN23042	CG9109	FBgn0031765	-	B3GLCT	0.49
BKN28870	CG6476	FBgn0003600	Suppressor of variegation 3-9	EHMT2, EHMT1, SUV39H1, SUV39H2, SE	0.49
BKN28397	CG6884	FBgn0036811	Mediator complex subunit 11	MED11	0.49
BKN40049	CG40351	FBgn0040022	SET domain containing 1	KMT2D, WHSC1L1, DIDO1, SETD2, SETD	0.49
BKN25766	CG5418	FBgn0032436	-	-	0.49
BKN41177	CG33873	FBgn0053873	His4:CG33873	HIST2H4B, HIST1H4A, HIST1H4E, HIST1	0.49
BKN45015	CG4173	FBgn0014029	Septin 2	SEPT9, SEPT12, SEPT5, SEPT4, SEPT10	0.50
BKN20682	CG6238	FBgn0029157	slingshot	DUSP26, DUSP2, DUPD1, STYX, DUSP2	0.50
BKN22139	CG6538	FBgn0010421	Transcription factor TFIIFbeta	GTF2F2	0.50
BKN33475	HDC07387		-	-	0.50
BKN31132	CG5151	FBgn0036576	-	LDLRAD4	0.50
BKN42696	CR33727	FBgn0053727	snmRNA:430:CR33727	-	0.50
BKN34337	HDC05416		-	-	0.50
BKN20488	CG17054	FBgn0259876	Chromosome associated protein G	NCAPG	0.50
BKN41212	CG33890	FBgn0053890	His2B:CG33890	HIST1H2BL, HIST2H2BE, HIST1H2BD, HI	0.50
BKN20863	CG9045	FBgn0002914	Myb oncogene-like	MYBL1, DMTF1, MYB, SNAPC4, MYBL2	0.50
BKN20912	CG8019	FBgn0001179	haywire	ERCC3	0.50
BKN29074	CG10377	FBgn0004838	Heterogeneous nuclear ribonucleoprotein at 27C	HNRNPA3, HNRNPA0, HNRNPA2B1, HNI	0.50
BKN22437	CG8464	FBgn0038233	HTRA2-related serine protease	HTRA1, NAGK, HTRA2, HTRA3, HTRA4,	0.50
BKN33743	HDC15018		-	-	0.50
BKN22921	CG5546	FBgn0036761	Mediator complex subunit 19	MED19	0.50
BKN27733	CG13671	FBgn0035867	-	PDIA2, PDIA3, TXNDC11, PDIA4, P4HB	0.50
BKN25466	CG15800	FBgn0034904	-	SKP1	0.50
BKN21853	CG9537	FBgn0031820	Daxx-like protein	DAXX	0.50
BKN24305	CG8213	FBgn0033359	-	HABP2, F12, HGF, CTRC, CELA1, CELA2	0.50
BKN26863	CG13942	FBgn0085408	Shroom	SHROOM2, SHROOM1, SHROOM3, SHR	0.50
BKN30994	CG15247	FBgn0030156	-	-	0.51
BKN45008	CG5949	FBgn0012066	DNA-polymerase-delta	POLD1, REV3L	0.51
BKN45506	CG5954	FBgn0002441	lethal (3) malignant brain tumor	L3MBTL4, SAMD1, SCML1, L3MBTL3, L3	0.51
BKN20729	CG30085	FBgn0050085	Rap1 interacting factor 1	RIF1	0.51
BKN31013	CG5134	FBgn0260401	Mediator complex subunit 9	MED9	0.51
BKN45406	CG11176	FBgn0030503	Transport and Golgi organization 2	TANGO2	0.51
BKN41298	CG43161	FBgn0037820	Skeletor	THBD	0.51
BKN24385	CG9985	FBgn0016984	skittles	PIP5K1B, PIP5K1A, PIP4K2C, PIP5KL1, P	0.51
BKN34093	HDC06513	FBgn0085215	-	-	0.51

BKN27586	CG8843	FBgn0031537	Secretory 5	EXOC2	0.51
BKN29324	CG18174	FBgn0028694	Regulatory particle non-ATPase 11	PSMD14, MPND, BRCC3, MYSM1	0.51
BKN41519	CG16988	FBgn0040291	Regulator of cullins 1b	RBX1, RNF7	0.51
BKN40893	CG34000	FBgn0054000	-	-	0.51
BKN42699	CR33740	FBgn0053740	snmRNA:430:CR33740	-	0.51
BKN31335	CG12362	FBgn0036082	-	CUL7, RNF19B, ARIH2, RNF31, ANKIB1,	0.52
BKN28401	CG7583	FBgn0020496	C-terminal Binding Protein	CTBP2, CTBP1, PHGDH	0.52
BKN30278	CG12387	FBgn0011556	zetaTrypsin	PRSS53, HPN, PRSS36, PRSS48	0.52
BKN45035	CG17139	FBgn0260454	-	VDAC1, VDAC2, VDAC3	0.52
BKN20759	CG31063	FBgn0051063	-	NUDT14	0.52
BKN29817	CG1999	FBgn0029947	-	-	0.52
BKN41327	CG33841	FBgn0053841	His2A:CG33841	HIST1H2AI, HIST1H2AG, H2AFJ, HIST1H	0.52
BKN23525	CG11821	FBgn0038680	Cyp12a5	CYP24A1, CYP11B1, CYP27A1, CYP11A	0.52
BKN27849	CG4677	FBgn0039039	lame duck	GLI1, GLIS1, GLI2, AAAS, GLIS3, GLI3, G	0.52
BKN20176	CG2503	FBgn0010750	antimeros	BBS5, PAF1, KLHL40	0.52
BKN22581	CG3727	FBgn0010583	dreadlocks	NCK2, NCK1	0.52
BKN42713	CR33739	FBgn0053739	snmRNA:430:CR33739	-	0.52
BKN24741	CG1644	FBgn0031182	Cyp6t1	CYP3A5, CYP3A4, CYP3A7-CYP3A51P, C	0.52
BKN20281	CG12225	FBgn0028982	Spt6	SUPT6H	0.52
BKN22369	CG8641	FBgn0035733	-	RERGL, DIRAS3, RALB, RAP2A, KRAS, F	0.52
BKN23635	CG8981	FBgn0260792	Microcephalin	MCPH1, BARD1	0.52
BKN27322	CG8749	FBgn0016978	small ribonucleoprotein particle U1 subunit 70K	RBM24, SNRNP35, SNRNP70	0.52
BKN41231	CG33904	FBgn0053904	His2B:CG33904	HIST1H2BG, HIST1H2BF, HIST1H2BA, HI	0.52
BKN30946	CG7917	FBgn0016685	Nucleoplasmin	NPM1, NPM2, NPM3	0.52
BKN31856	CG14821	FBgn0035719	target of wingless	C1orf21	0.53
BKN27273	CG15631	FBgn0031626	-	-	0.53
BKN30209	CG3857	FBgn0023520	-	SMG9	0.53
BKN31271	CG31157	FBgn0051157	-	TMEM135	0.53
BKN25853	CG9733	FBgn0039759	-	-	0.53
BKN30340	CG15186	FBgn0037448	-	-	0.53
BKN20724	CG10480	FBgn0002638	Regulator of chromosome condensation 1	SERGEF, NEK9, RCC1, RCC1L, RCBTB1	0.53
BKN27711	CG8597	FBgn0011640	lark	RBM4B, ADK, RBM14-RBM4, HNRNPCL1	0.53
BKN42004	CG14421	FBgn0029644	-	-	0.53
BKN24231	CG6168	FBgn0036154	-	QPCT, QPCTL	0.53
BKN22761	CG4157	FBgn0028693	Regulatory particle non-ATPase 12	PSMD8	0.53
BKN30821	CG15034	FBgn0029946	-	-	0.53
BKN46692	CG13862	FBgn0038942	-	-	0.53
BKN46094	CG8025	FBgn0038460	Mtr3	EXOSC4, EXOSC6	0.53
BKN22485	CG12373	FBgn0026741	mitochondrial ribosomal protein L18	MRPL18	0.53
BKN22766	CG5800	FBgn0030855	-	DDX18, DDX10, KCTD19	0.53
BKN20476	CG14788	FBgn0028274	Nucleostemin 3	GNL1, LSG1	0.53
BKN29607	CG6631	FBgn0039206	anastral spindle 1	-	0.53
BKN28296	CG6896	FBgn0036801	MYPT-75D	PPP1R12B, NFKBIE, PPP1R12C, PPP1R1	0.53
BKN45761	CG18622	FBgn0036916	-	-	0.53
BKN27305	CG6050	FBgn0024556	mitochondrial translation elongation factor Tu 1	TUFM	0.53
BKN20600	CG9191	FBgn0004378	Kinesin-like protein at 61F	KIF11, KIF3A	0.54
BKN31100	CG31330	FBgn0086901	crossveinless c	ARHGAP18, ARHGAP11A, STARD13, AR	0.54
BKN45103	CG14749	FBgn0033316	-	GLE1	0.54
BKN45613	CG6175	FBgn0036152	-	-	0.54
BKN45523	CG42276	FBgn0259171	Phosphodiesterase 9	PDE4D, PDE10A, PDE2A, PDE8B, PDE4C	0.54
BKN21536	CG4063	FBgn0023444	ebi	TBL1Y, WDR38, TBL1X, TBL1XR1	0.54
BKN21248	CG5179	FBgn0019949	Cyclin-dependent kinase 9	CDK13, CDK12, CDK9	0.54
BKN27526	CG9968	FBgn0030749	Annexin B11	ANXA2, ANXA4, ANXA13, ANXA6, ANXA7	0.54
BKN21895	CG12343	FBgn0033556	-	SYF2	0.54
BKN22574	CG12245	FBgn0014179	glial cells missing	GCM2, GCM1	0.54
BKN23852	CG12885	FBgn0039523	-	-	0.54
BKN20845	CG5170	FBgn0027835	Dodeca-satellite-binding protein 1	HDLBP, BICC1	0.54
BKN20745	CG7274	FBgn0085451	hat-trick	RBM34, ARID2, ARID5B, ARID3A, ARID3E	0.54
BKN20490	CG11337	FBgn0039846	Polynucleotide phosphorylase	PNPT1	0.54
BKN46638	CG2674	FBgn0005278	S-adenosylmethionine Synthetase	MAT2A, MAT1A	0.54
BKN29446	CG15862	FBgn0022382	Protein kinase, cAMP-dependent, regulatory subunit t	PRKAR2B, CNBD2, PRKAR1B, PRKAR2A	0.54
BKN21411	CG15319	FBgn0015624	nejire	CREBBP, EP300	0.54

BKN25453	CG2989	FBgn0030171	Chitinase 6	SRL, OVGP1, CHIT1, CTBS, CHI3L1, CHI	0.54
BKN32559	CG33323	FBgn0037475	48 related 1	NHLH1, LYL1, FIGLA, SCX, ASCL5, MSC,	0.54
BKN23069	CG14085	FBgn0036859	-	KIAA0825	0.54
BKN40736	CR32481	FBgn0052481	transfer RNA:Isoleucine-TAT 1-2	-	0.54
BKN20329	CG8542	FBgn0001220	Heat shock protein cognate 5	HSPA9	0.54
BKN46664	CG34422	FBgn0085451	hat-trick	RBM34, ARID2, ARID5B, ARID3A, ARID3I	0.55
BKN29139	CG3034	FBgn0040339	Mediator complex subunit 22	MED22	0.55
BKN27496	CG7943	FBgn0039741	-	SLC25A52, SLC25A53, SLC25A51	0.55
BKN32372	CG13937	FBgn0035287	-	CHST12, CHST8, CHST11, CHST13, CHS	0.55
BKN20388	CG1341	FBgn0028687	Regulatory particle triple-A ATPase 1	WDR12, PSMC2, PSMC5, PSMC6	0.55
BKN27734	CG5281	FBgn0037902	-	SLC35G4, SLC35G1, SLC35G3, SLC35G	0.55
BKN40482	CG18743	FBgn0013276	Heat-shock-protein-70Ab	HSPA1B, NCKAP1L, HSPA8, HSPA12A, H	0.55
BKN40104	CG13330	FBgn0033848	-	-	0.55
BKN20542	CG9752	FBgn0034614	-	C9orf64	0.55
BKN32123	CG11768	FBgn0037625	-	ACTC1	0.55
BKN27493	CG7614	FBgn0024956	Menage a trois 1	MNAT1	0.55
BKN28845	CG1057	FBgn0037262	Mediator complex subunit 31	MED31	0.55
BKN46250	CG7137	FBgn0034422	-	RRP8	0.55
BKN33034	CG6449	FBgn0036101	Ninjurin A	NINJ2, NINJ1	0.55
BKN34301	HDC06747		-	-	0.55
BKN33914	HDC02355		-	-	0.55
BKN25748	CG10704	FBgn0036285	twin of eyg	GBX1, PAX1, DUX4, PAX4, DUXA, PAX2,	0.56
BKN20700	CG15160	FBgn0032688	-	RPRD1B, RPRD2, RPRD1A	0.56
BKN22484	CG5371	FBgn0011703	Ribonucleoside diphosphate reductase large subunit	RRM1	0.56
BKN20516	CG7940	FBgn0038576	Actin-related protein 5	ACTR5	0.56
BKN20392	CG17800	FBgn0033159	Down syndrome cell adhesion molecule 1	PTPRU, MYLK4, PTPRM, DSCAML1, IL12	0.56
BKN27429	CG5041	FBgn0031309	Transcription factor B4	GTF2H3	0.56
BKN21141	CG32082	FBgn0052082	Insulin receptor substrate 53 kDa	ALG10, PACSIN3, ALG10B, BAIAP2L2, B/	0.56
BKN29599	CG9476	FBgn0003886	alpha-Tubulin at 85E	TUBA1B, TUBA3E, TUBA1A, TUBAL3, TU	0.56
BKN41322	CG33826	FBgn0053826	His2A:CG33826	HIST1H2AB, H2AFB2, H2AFB3, HIST2H2/	0.56
BKN21616	CG4200	FBgn0003416	small wing	PLCG1, PLCD3, PLCL2, PLCD1, PLCD4,	0.56
BKN22862	CG12113	FBgn0026679	Integrator 4	INTS4	0.56
BKN30922	CG31466	FBgn0051466	-	-	0.56
BKN45655	CG8484	FBgn0037751	matotopetli	ZNF543, ZBTB24, ZNF331, ZNF785	0.56
BKN30941	CG9775	FBgn0037261	-	CHTOP	0.56
BKN21555	CG10484	FBgn0000486	Regulatory particle non-ATPase 3	PSMD3	0.56
BKN23374	CG7903	FBgn0039730	-	RBM4, ADK, RBM14	0.56
BKN22677	CG4013	FBgn0024308	Smrter	PRRC2C, NCOR2, NCOR1	0.56
BKN31382	CG12172	FBgn0024294	Serpin 43Aa	SERPINA10, SERPINE3, SERPINA5, SER	0.56
BKN32750	CG31109	FBgn0051109	-	-	0.56
BKN42213	CG34232	FBgn0085261	-	-	0.56
BKN24585	CG4701	FBgn0028868	-	ATAD1	0.56
BKN26233	CG5430	FBgn0011294	antennal protein 5	MRPL38, PEBP4, PEBP1	0.56
BKN27646	CG7977	FBgn0026372	Ribosomal protein L23A	RPL23A	0.56
BKN28335	CG5545	FBgn0032651	Olig family	OLIG3, OLIG1, OLIG2, BHLHE23, BHLHE	0.56
BKN25762	CG14625	FBgn0040358	-	-	0.56
BKN21376	CG5454	FBgn0038667	small ribonucleoprotein particle U1 subunit C	SNRPC, ZMAT5	0.57
BKN45675	CG43745	FBgn0035330	Myosuppressin receptor 2	GPR139, GPR142	0.57
BKN45701	CG3820	FBgn0010660	Nucleoporin 214kD	NUP214	0.57
BKN24658	CG17083	FBgn0039070	-	CCDC114, CCDC63	0.57
BKN20647	CG4561	FBgn0027080	Tyrosyl-tRNA synthetase	YARS, AIMP1	0.57
BKN41561	CG33857	FBgn0053857	His3:CG33857	HIST1H3E, HIST2H3C, H3F3C, H3F3A, H	0.57
BKN46374	CG42783	FBgn0260478	atypical protein kinase C	PRKCZ, PRKCI	0.57
BKN29679	CG5175	FBgn0038476	kugelkern	-	0.57
BKN42300	CR34530	FBgn0082993	snoRNA:Psi28S-1936	-	0.57
BKN25849	CG3986	FBgn0022700	Chitinase 4	CHIT1, CHI3L2, SRL, CHI3L1, OVGP1, CH	0.57
BKN46507	CG13837	FBgn0039042	-	-	0.57
BKN33336	CG11668	FBgn0038113	-	F10, MASP1, PROC, F7	0.57
BKN29134	CG14903	FBgn0038446	-	PTRHD1	0.57
BKN30796	CG13936	FBgn0035282	CNMamide	-	0.57
BKN21818	CG4184	FBgn0027592	Mediator complex subunit 15	MED15	0.57
BKN29989	CG10591	FBgn0035621	-	-	0.57

BKN21805	CG7393	FBgn0024846	p38b MAP kinase	MAPK7, MAPK12, MAPK1, MAPK14, MAF	0.57
BKN29457	CG5226	FBgn0034355	-	SLC6A19, SLC6A4, SLC6A5, SLC6A14, S	0.57
BKN20837	CG8583	FBgn0035771	Secretory 63	SEC63	0.57
BKN45135	CG12323	FBgn0029134	Proteasome beta5 subunit	PSMB8, PSMB11, PSMB5	0.57
BKN22072	CG4097	FBgn0002284	Proteasome beta6 subunit	PSMB1	0.57
BKN40607	CG34315	FBgn0085344	-	-	0.57
BKN24387	CG11798	FBgn0015371	charlatan	-	0.57
BKN21254	CG7764	FBgn0036513	marionette	GTF2H4	0.57
BKN30129	CG3569	FBgn0034865	Odorant receptor 59b	-	0.57
BKN32308	CG10320	FBgn0034645	NADH dehydrogenase (ubiquinone) B12 subunit	NDUFB3	0.57
BKN46087	CG9468	FBgn0032069	Lysosomal alpha-mannosidase VI	MAN2B1, MAN2A1, MAN2B2, MAN2A2	0.57
BKN20168	CG4202	FBgn0029755	Something about silencing 10	UTP3	0.58
BKN46032	CG42309	FBgn0259209	Muscle LIM protein at 60A	CSRP2, CSRP1, CSRP3, CRIP1	0.58
BKN27691	CG5121	FBgn0039337	Mediator complex subunit 28	MED28, ALKBH6	0.58
BKN41108	CG40042	FBgn0058042	Translocase of inner mitochondrial membrane 23	TIMM23B, TIMM23	0.58
BKN29556	CG33464	FBgn0053464	slit	SLIT2, VASN, ECM2, LRRC3C, LRRC3B,	0.58
BKN22791	CG14220	FBgn0031036	-	BRMS1, BRMS1L, SUDS3	0.58
BKN45430	CG31155	FBgn0051155	Rpb7	POLR2G	0.58
BKN29185	CG5986	FBgn0043455	-	SDE2	0.58
BKN31636	CG17637	FBgn0037004	-	SLC46A1, SLC46A2, MFSD14B, SLC46A3	0.58
BKN20813	CG10361	FBgn0036208	-	GCAT, ALAS1, NCLN, ALAS2	0.58
BKN46408	CG42273	FBgn0039345	minibrain	DYRK4, DYRK3, DYRK2, DYRK1A, HIPK1	0.58
BKN21063	CG7886	FBgn0038248	-	NOD2, LRRC71, LRRC74A, CEP78, NOD	0.58
BKN29963	CG11370	FBgn0037181	-	-	0.58
BKN27091	CG8374	FBgn0016792	dalmatian	-	0.58
BKN21944	CG7382	FBgn0031708	-	TIMM21	0.58
BKN32279	CG15211	FBgn0030234	-	CKLF, CKLF-CMTM1, CMTM3, MAL, CMT	0.58
BKN20786	CG3423	FBgn0020616	Stromalin	STAG3, STAG1, STAG2	0.58
BKN32000	CG14952	FBgn0035377	-	-	0.58
BKN20178	CG15899	FBgn0029846	Ca[2+]-channel protein alpha[[1]] subunit T	CACNA1G, CATSPER1, CACNA1I, CACN	0.58
BKN21117	CG5222	FBgn0036570	Integrator 9	INTS9	0.58
BKN45829	CG5476	FBgn0039441	TweedleN	-	0.58
BKN26096	CG3528	FBgn0031430	-	C4orf22	0.58
BKN26496	CG31562	FBgn0051562	-	-	0.58
BKN28256	CG17331	FBgn0032596	Proteasome beta4 subunit	PSMB2	0.58
BKN20746	CG8545	FBgn0033741	-	NOP2, NSUN6	0.58
BKN24944	CG7065	FBgn0030091	-	SMARCA4, SMARCA2	0.59
BKN21843	CG4252	FBgn0004367	meiotic 41	ATR	0.59
BKN20213	CG10809	FBgn0036052	-	ANKRD46, ANKRD61, ANKRD54	0.59
BKN41308	CG33820	FBgn0053820	His2A:CG33820	HIST3H2A, H2AFJ, HIST2H2AC, HIST1H2	0.59
BKN31089	CG8192	FBgn0034030	-	-	0.59
BKN20527	CG8190	FBgn0034029	eukaryotic translation initiation factor 2B subunit gamı	EIF2B3	0.59
BKN45433	CG9386	FBgn0037708	-	TRMT44	0.59
BKN32651	CG14490	FBgn0034281	-	MORN3, RSPH10B, MORN4, ALS2CL, JP	0.59
BKN21205	CG1640	FBgn0030478	-	GPT2, GPT	0.59
BKN26525	CG33129	FBgn0053129	-	TMEM214	0.59
BKN20077	CG4800	FBgn0037874	Translationally controlled tumor protein	FLJ44635, TPT1	0.59
BKN41321	CG33823	FBgn0053823	His2A:CG33823	HIST1H2AB, H2AFJ, HIST1H2AG, HIST1H	0.59
BKN22874	CG30077	FBgn0050077	Biogenesis of lysosome-related organelles complex 1	BLOC1S1	0.59
BKN32652	CG32536	FBgn0052536	-	EBAG9	0.59
BKN29194	CG1417	FBgn0003423	sluggish A	PRODH2, LOC102724788, PRODH	0.59
BKN25295	CG15654	FBgn0085425	-	KCNK4, KCNK18, KCNK1, KCNK17, KCNI	0.59
BKN41319	CG42342	FBgn0000320	-	COL5A1, COL17A1, COL6A3, COL14A1, (	0.59
BKN21440	CG5188	FBgn0032247	-	METAP1, METAP1D	0.59
BKN41497	CG12546	FBgn0037178	-	-	0.59
BKN45099	CG10573	FBgn0020294	knockout	STOX1, STOX2	0.59
BKN22886	CG9998	FBgn0005411	U2 small nuclear riboprotein auxiliary factor 50	U2AF2, UHMK1	0.59
BKN21608	CG15087	FBgn0034380	Vacuolar protein sorting 51	VPS51	0.59
BKN26746	CG7293	FBgn0004381	Kinesin-like protein at 68D	KIF3C, KIF5A, KIF5B, KIF3B, KIF5C, KIFC	0.59
BKN32250	CG4022	FBgn0035986	-	PIH1D2	0.59
BKN41734	CG34320	FBgn0085349	-	-	0.59
BKN45279	CG30419	FBgn0050419	-	-	0.59

BKN40615	CR31512	FBgn0011999	transfer RNA:Valine-CAC 2-1	-	0.60
BKN25674	CG32091	FBgn0052091	-	ABCG1, ABCG4, ABCG5	0.60
BKN29964	CG5717	FBgn0041709	yellow-g	DPM3	0.60
BKN28865	CG16890	FBgn0028932	-	FRA10AC1	0.60
BKN27669	CG17754	FBgn0030114	-	KLHL3, KLHL12, KLHL4, KBTBD11, KLHD	0.60
BKN40699	CG9987	FBgn0032781	RtcB-like	RTCB	0.60
BKN22938	CG17337	FBgn0259979	-	CNDP1, CNDP2	0.60
BKN41442	CG32037	FBgn0052037	-	-	0.60
BKN23561	CG31630	FBgn0051630	milton	HAP1, TRAK1, TRAK2	0.60
BKN41107	CG11051	FBgn0040813	Neuropeptide-like precursor 2	-	0.60
BKN41930	CG32069	FBgn0052069	-	IER3IP1, HDHD2, LHPP	0.60
BKN27987	CG4904	FBgn0250843	Proteasome alpha6 subunit	PSMA1	0.60
BKN25456	CG33456	FBgn0085444	muscle wasted	GON4L, YY1AP1	0.60
BKN22605	CG3284	FBgn0004855	RNA polymerase II 15kD subunit	POLR2I	0.60
BKN21781	CG10223	FBgn0003732	Topoisomerase 2	TOP2B, TOP2A	0.60
BKN41159	CG34230	FBgn0085259	-	-	0.60
BKN31383	CG5650	FBgn0004103	Protein phosphatase 1 at 87B	PPP1CC, PPP1CA, PPP1CB	0.60
BKN42643	CG12405	FBgn0033520	Peroxiredoxin 2540-1	PRDX3, PRDX4, PRDX6, PRDX1, PRDX2	0.60
BKN21118	CG9633	FBgn0010173	Replication Protein A 70	RPA1	0.60
BKN31925	CG32693	FBgn0052693	Gustatory receptor 9a	-	0.60
BKN30049	CG15738	FBgn0030352	severe impairment of CI with lengthened youth	NDUFAF6	0.60
BKN41085	CG44328	FBgn0052635	Neuropilin and tolloid-like	NETO1, NETO2	0.60
BKN23328	CG13455	FBgn0036498	-	-	0.60
BKN27655	CG10978	FBgn0037374	jagunal	JAGN1	0.60
BKN40674	CG6806	FBgn0002565	Larval serum protein 2	-	0.60
BKN40550	CG40858	FBgn0085527	-	-	0.61
BKN26034	CG14907	FBgn0038455	-	-	0.61
BKN28882	CG7808	FBgn0039713	Ribosomal protein S8	RPS8	0.61
BKN26486	CG7669	FBgn0038607	hemingway	CFAP97	0.61
BKN23689	CG17264	FBgn0031490	-	-	0.61
BKN28504	CG3668	FBgn0004896	forkhead domain 59A	FOXC2, FOXB1, FOXS1, FOXB2, FOXA1,	0.61
BKN31813	CG3474	FBgn0028871	Cuticular protein 35B	-	0.61
BKN23975	CG12327	FBgn0036492	Bestrophin 3	BEST4, BEST1, BEST3, BEST2	0.61
BKN27133	CG14630	FBgn0014903	-	TMLHE, BBOX1	0.61
BKN24094	CG14416	FBgn0040352	-	-	0.61
BKN33712	HDC12227		-	-	0.61
BKN24498	CG8644	FBgn0038133	Osiris 22	-	0.61
BKN26869	CG5669	FBgn0039169	Sp1-like factor for pairing sensitive-silencing	SP9, SP4, SP2, SP5, SP8, KLF10, SP3, S	0.61
BKN41306	CG33814	FBgn0053814	His2A:CG33814	HIST1H2AE, HIST2H2AB, HIST1H2AK, HI	0.61
BKN30911	CG14177	FBgn0036013	Transmembrane channel-like	TMC8, TMC2, TMC4, TMC1, TMC7, TMC5	0.61
BKN27784	CG6708	FBgn0020626	Oxysterol binding protein	OSBPL3, OSBPL7, OSBP, OSBP2, OSBP	0.61
BKN30844	CG15204	FBgn0259241	-	SBSPON	0.61
BKN22457	CG16728	FBgn0033539	G protein-coupled receptor kinase interacting ArfGAP	GIT1, AGAP2, GIT2	0.61
BKN25186	CG33519	FBgn0053519	Unc-89	PLEKHG4B, PSKH1, HMCN2, TTN, CAMK	0.61
BKN20484	CG10198	FBgn0039120	Nucleoporin 98-96kD	NUP58, NUP98, NCOA5, NUP62	0.61
BKN24588	CG4323	FBgn0038797	Dicarboxylate carrier 2	UCP2, SLC25A10, UCP1, UCP3, SLC25A	0.61
BKN24987	CG7384	FBgn0032262	-	-	0.61
BKN25452	CG4757	FBgn0027584	-	CES3, NLGN4Y, NLGN2, CEL, NLGN4X, (	0.61
BKN27217	CG31177	FBgn0259237	-	TRHDE, ERAP2, ANPEP, LNPEP, NPEPP	0.61
BKN30350	CG32150	FBgn0052150	-	RASSF7, RASSF8, RASSF9, RASSF10	0.61
BKN42613	CG16824	FBgn0040973	-	TMA7	0.61
BKN27199	CG8902	FBgn0031886	Nuf2	CCDC39	0.61
BKN20389	CG10697	FBgn0000422	Dopa decarboxylase	HDC, DDC	0.61
BKN27246	CG2714	FBgn0000520	cramped	CRAMP1	0.61
BKN23032	CG9590	FBgn0038360	-	FAM114A2, FAM114A1	0.61
BKN26556	CG5740	FBgn0259736	-	-	0.61
BKN21463	CG2469	FBgn0035205	Ctr9	CTR9	0.62
BKN42185	CG34322	FBgn0085351	inaF-A	-	0.62
BKN41205	CG6824	FBgn0003028	ovo	OVOL3, ZNF770, OVOL2, ZNF131, REST,	0.62
BKN41423	CG40189	FBgn0058189	-	-	0.62
BKN28477	CG4665	FBgn0035964	Dihydropteridine reductase	QDPR	0.62
BKN21625	CG14299	FBgn0038651	ectopic P-granules autophagy protein 5	EPG5	0.62

BKN25718	CG8179	FBgn0034020	-	STAC2, STAC3, STAC	0.62
BKN26829	CG14273	FBgn0032024	-	-	0.62
BKN32404	CG33936	FBgn0053936	-	ZFAND6, ZFAND5	0.62
BKN30160	CG8348	FBgn0012344	Diuretic hormone 44	-	0.62
BKN30900	CG9819	FBgn0030758	Calcineurin A at 14F	PPP3CA, PPP3CB, PPP3CC	0.62
BKN45051	CG8503	FBgn0033917	SET and MYND domain containing, arthropod-specifi	SMYD4, SMYD2, ZMYND15, SMYD1, SM	0.62
BKN27418	CG10947	FBgn0032857	-	CAMKMT	0.62
BKN21446	CG15523	FBgn0039727	Vacuolar protein sorting 13B	VPS13B, VPS13A, VPS13D, VPS13C	0.62
BKN46196	CG9131	FBgn0029161	slowmo	PRELID3A, PRELID3B, PRELID2	0.62
BKN31215	CG8929	FBgn0034504	-	C11orf57, SRRM4	0.62
BKN26102	CG9487	FBgn0085427	-	-	0.62
BKN25494	CG9512	FBgn0030593	-	CHDH	0.62
BKN25727	CG33202	FBgn0053202	defective proboscis extension response 11	KIRREL, KIRREL3, IGSF9B, KIRREL2, US	0.62
BKN46371	CG10270	FBgn0022699	D19B	ZNF341, ZNF668, ZNF30, ZNF852, ZNF2	0.62
BKN45552	CG8749	FBgn0016978	small ribonucleoprotein particle U1 subunit 70K	RBM24, SNRNP35, SNRNP70	0.62
BKN24587	CG17186	FBgn0038741	-	-	0.62
BKN33798	HDC18361		-	-	0.62
BKN22389	CG4893	FBgn0036616	ringmaker	TPPP, TPPP3, TPPP2	0.62
BKN31788	CG30267	FBgn0050267	-	-	0.62
BKN22082	CG31671	FBgn0031390	tho2	THOC2, ZC3H13	0.62
BKN33975	HDC12388	FBgn0260723	-	-	0.62
BKN25694	CG33455	FBgn0085444	muscle wasted	GON4L, YY1AP1	0.62
BKN29054	CG11290	FBgn0034975	enoki mushroom	KAT7, KAT5, KAT8, KAT6B, KAT6A	0.62
BKN46483	CG12907	FBgn0250840	-	-	0.63
BKN25668	CG13443	FBgn0034548	-	-	0.63
BKN25003	CG4936	FBgn0038768	-	ZNF730, ZNF792, ZNF732, ZSCAN2, ZNF	0.63
BKN21627	CG7671	FBgn0038609	Nucleoporin 43kD	NUP43	0.63
BKN25863	CG11092	FBgn0027537	Nucleoporin 93kD-1	NUP93	0.63
BKN41490	CG34448	FBgn0085477	-	LIPI, PNLIPRP2, LIPH, PNLIPRP3, PLA1A	0.63
BKN23972	CG9498	FBgn0031801	-	-	0.63
BKN41257	CG33876	FBgn0053876	His2B:CG33876	HIST3H2BB, HIST2H2BF, H2BFM, H2BFW	0.63
BKN20648	CG9143	FBgn0034496	-	DDX24	0.63
BKN24209	CG10142	FBgn0035076	Ance-5	TMEM27, ACE2, ACE	0.63
BKN22288	CG8787	FBgn0000142	Additional sex combs	ASXL1, ASXL3, ASXL2	0.63
BKN27426	CG12085	FBgn0028577	half pint	PUF60	0.63
BKN22200	CG14716	FBgn0037933	Heme oxygenase	HMOX2, HMOX1	0.63
BKN24592	CG6061	FBgn0033846	Myb-interacting protein 120	LIN54, TESMIN	0.63
BKN29977	CG4491	FBgn0005771	no ocelli	ZNF503, ZNF703	0.63
BKN27538	CG18009	FBgn0026758	TATA box binding protein-related factor 2	TBPL1, TBPL2, TBP	0.63
BKN41325	CG33835	FBgn0053835	His2A:CG33835	H2AFB3, H2AFJ, HIST1H2AB, H2AFY, HI	0.63
BKN21787	CG5018	FBgn0036578	lethal (3) 72Dn	UTP4	0.63
BKN21514	CG6015	FBgn0038927	-	CDC40	0.63
BKN20776	CG10691	FBgn0002031	lethal (2) 37Cc	PHB, PHB2	0.63
BKN22828	CG2128	FBgn0025825	Histone deacetylase 3	HDAC3, HDAC1, HDAC2, HDAC8	0.63
BKN27279	CG30339	FBgn0050339	-	CLVS1, SEC14L1, CLVS2, SEC14L5, SEC	0.63
BKN28990	CG7398	FBgn0024921	Transportin	TNPO1, TNPO2	0.63
BKN30252	CG3241	FBgn0005616	male-specific lethal 2	MSL2	0.63
BKN28409	CG2910	FBgn0027548	spenito	SPEN, RBM15, RBM15B	0.63
BKN25836	CG7257	FBgn0036224	Regulatory particle triple-A ATPase 4-related	PSMC6, PSMC2, WDR12, PSMC5	0.63
BKN31798	CG14405	FBgn0032888	Chemosensory protein B 38c	-	0.63
BKN20650	CG16984	FBgn0062517	-	ENKUR	0.63
BKN31014	CG8867	FBgn0020906	Jonah 25Bi	KLK3	0.63
BKN27649	CG4528	FBgn0003449	sans fille	SNRPA, RNPC3, SNRPB2	0.63
BKN45569	CG7756	FBgn0001217	Heat shock protein cognate 2	HSPA1A, HSPA6, HSPA1B, HSPA5, HSP	0.63
BKN26871	CG2985	FBgn0004045	Yolk protein 1	LIPC, LPL, PLA1A, PNLIPRP1, LIPI, LIPH,	0.64
BKN32070	CG3379	FBgn0013981	Histone H4 replacement	HIST1H4B, HIST1H4A, HIST4H4, HIST1H	0.64
BKN21455	CG12323	FBgn0029134	Proteasome beta5 subunit	PSMB8, PSMB11, PSMB5	0.64
BKN21968	CG7840	FBgn0032014	-	SRD5A3	0.64
BKN26282	CG32628	FBgn0052628	Nna1 carboxypeptidase	AGBL3, AGBL5, AGBL2, AGTPBP1, AGBL	0.64
BKN22618	CG5602	FBgn0034922	DNA ligase I	LIG1	0.64
BKN27762	CG3328	FBgn0034985	-	MYRFL, MYRF	0.64
BKN20532	CG9900	FBgn0004643	Zeste-white 10	ZW10	0.64

BKN32368	CG31051	FBgn0051051	-	-	0.64
BKN27830	CG14514	FBgn0039654	Bromodomain containing 8	BRD8	0.64
BKN26108	CG6265	FBgn0039478	Neprilysin 5	ECEL1, MMEL1, KEL, PHEX, ECE1, ECE2	0.64
BKN33601	HDC09484		-	-	0.64
BKN23274	CG33509	FBgn0053509	-	-	0.64
BKN32486	CG15048	FBgn0030935	Beadex	LMO3, LMO2, LMO1, LMO4	0.64
BKN24575	CG11608	FBgn0038069	-	LIPK, LIPJ, LIPF, LIPN, LIPM, LIPA	0.64
BKN21026	CG6443	FBgn0032290	-	RTFDC1	0.64
BKN34674	HDC20621	FBgn0058042	-	-	0.64
BKN27845	CG3918	FBgn0029873	-	SREK1IP1, FAM133B	0.64
BKN23390	CG8959	FBgn0030691	EF-hand domain containing 1.1	EFHC1, EFHC2	0.64
BKN30037	CG13964	FBgn0032831	sickie	NAV1, NAV2, NAV3	0.64
BKN25780	CG5436	FBgn0001230	Heat shock protein 68	HSPA8, HSPA5, HSPA12B, HSPA6, NCKA	0.64
BKN21817	CG7556	FBgn0030990	-	DNAJC7, DNAJC1, DNAJC4, DNAJC18	0.64
BKN29888	CG13364	FBgn0026879	-	TMA7	0.64
BKN31402	CG3183	FBgn0033081	geminin	GMNC, MCIDAS, GMNN	0.64
BKN21478	CG6938	FBgn0036235	-	ANO9, ANO1, ANO4, ANO5, ANO2, ANO8	0.64
BKN20768	CG6323	FBgn0039465	Tetraspanin 97E	TSPAN13, TSPAN31	0.64
BKN22580	CG30100	FBgn0050100	-	MTRF1L, C12orf65, MTRF1	0.64
BKN46142	CG12118	FBgn0030101	-	MMADHC	0.64
BKN31114	CG1330	FBgn0004779	Ccp84Ae	-	0.65
BKN26941	CG10862	FBgn0035455	-	UBE2E1, UBE2N, UBE2D2, UBE2D4, UBE	0.65
BKN21879	CG10370	FBgn0028684	Regulatory particle triple-A ATPase 5	PSMC3	0.65
BKN22805	CG5363	FBgn0004106	Cyclin-dependent kinase 1	CDK5, CDK1, CDK18, CDK14, CDK3, CDK	0.65
BKN40571	CG12608	FBgn0030630	-	PAK1IP1, WDR55	0.65
BKN41539	CG33812	FBgn0053812	His3:CG33812	H3F3A, HIST1H3B, H3F3B, CENPA, HIST	0.65
BKN29520	CG31695	FBgn0005590	screw	GDF15, BMP6, TGFB2, TGFB3, INHBE, B	0.65
BKN22940	CG13630	FBgn0039219	-	METAP1D, METAP1	0.65
BKN31011	CG10788	FBgn0010295	new glue 3	-	0.65
BKN23037	CG3127	FBgn0250906	Phosphoglycerate kinase	PGK2, PGK1	0.65
BKN46080	CG15119	FBgn0034430	Myb-interacting protein 40	SMIM4, LIN37, C12orf73	0.65
BKN22014	CG16983	FBgn0025637	SKP1-related A	SKP1	0.65
BKN21760	CG12512	FBgn0031703	-	ACSF3, ACSF2	0.65
BKN20447	CG12084	FBgn0043458	-	ZYG11A, ZYG11B, ZER1	0.65
BKN33210	CG3327	FBgn0020445	Early gene at 23	ABCG8, ABCG5, ABCD4, ABCG4, ABCG1	0.65
BKN32545	CG13879	FBgn0035120	wee Augmin	-	0.65
BKN26249	CG9948	FBgn0035721	-	-	0.65
BKN27326	CG9131	FBgn0029161	slowmo	PRELID3A, PRELID3B, PRELID2	0.65
BKN33037	CG5084	FBgn0034288	-	-	0.65
BKN29130	CG4645	FBgn0030435	-	YIPF1, YIPF2	0.65
BKN27301	CG4938	FBgn0021750	Seryl-tRNA synthetase, mitochondrial	SARS2, FBXO17, SARS	0.65
BKN20695	CG11840	FBgn0031260	Signal peptide peptidase	SPPL2C, SPPL3, HM13	0.65
BKN41331	CG33853	FBgn0053853	His2A:CG33853	H2AFX, HIST1H2AD, H2AFY, HIST1H2AJ	0.65
BKN32342	CG15113	FBgn0004572	5-hydroxytryptamine (serotonin) receptor 1B	ADRA2A, HTR1E, HTR5A, HTR1D, HTR7,	0.65
BKN28143	CG9748	FBgn0000171	belle	DDX3Y, DDX3X, DDX4	0.65
BKN21637	CG4894	FBgn0001991	Ca[2+]-channel protein alpha[[1]] subunit D	SCN10A, CACNA1F, CACNA1S, CACNA1	0.65
BKN28430	CG9412	FBgn0015778	rasputin	G3BP2, G3BP1	0.65
BKN28091	CG12104	FBgn0035238	-	TOX3, UBTF, TOX, HMGB3, TOX4, TFAM	0.65
BKN40028	CG17139	FBgn0260453	-	VDAC1, VDAC2, VDAC3	0.66
BKN41013	CG34010	FBgn0054010	-	GLYAT, GLYATL1, GLYATL3, GLYATL2	0.66
BKN33326	CG33752	FBgn0053752	-	-	0.66
BKN28365	CG12000	FBgn0250746	Proteasome beta7 subunit	PSMB4	0.66
BKN26737	CG18212	FBgn0038535	aluminum tubes	RRBP1	0.66
BKN21325	CG3542	FBgn0031492	-	PRPF40B, PRPF40A	0.66
BKN29903	CG32813	FBgn0052813	-	RAD54B	0.66
BKN25182	CG17382	FBgn0039080	Ionotropic receptor 94h	GRIK5, GRID2, GRID1, GRIK3, GRIK2	0.66
BKN29148	CG2852	FBgn0034753	-	NKTR, PPIB, RGPD4, RGPD5, PPIAL4A, I	0.66
BKN22345	CG6769	FBgn0030878	-	ZNF622	0.66
BKN29089	CG5266	FBgn0086134	Proteasome alpha2 subunit	PSMA2, PSMA5, PSMA7, PSMA8	0.66
BKN27591	CG11295	FBgn0013548	lethal-(2)-denticleless	DTL	0.66
BKN29452	CG4147	FBgn0001218	Heat shock 70-kDa protein cognate 3	HSPA5, HSPA1L, HSPA14, HSPA8, HSPA	0.66
BKN21313	CG1749	FBgn0030305	Ubiquitin activating enzyme 5	UBA5	0.66

BKN20686	CG7292	FBgn0038269	Rrp6	EXOSC10	0.66
BKN41330	CG33850	FBgn0053850	His2A:CG33850	HIST1H2AH, HIST3H2A, HIST1H2AD, HIS	0.66
BKN41125	CG32825	FBgn0062565	Odorant receptor 19b	-	0.66
BKN26443	CG18417	FBgn0035780	-	CPA1, CPA3, CPA4, CPA5, CPA6, CPB2,	0.66
BKN25302	CG10911	FBgn0034295	-	-	0.66
BKN27597	CG8979	FBgn0033669	Proteasome inhibitor 31 kDa	PSMF1	0.66
BKN25047	CG1647	FBgn0039602	-	OVOL2, ZNF770, RP1, ZNF655, DMP1, IB	0.66
BKN45751	CG31666	FBgn0086758	Chronologically inappropriate morphogenesis	ZBTB12, ZBTB49, ZBTB9, ZBTB10, BACH	0.66
BKN23431	CG7532	FBgn0028915	lethal (2) 34Fc	FRRS1	0.66
BKN45159	CG11221	FBgn0031855	-	SBK1, SBK2, SBK3	0.66
BKN26559	CG8300	FBgn0029937	-	FAM177B, FAM177A1	0.66
BKN21730	CG10726	FBgn0014127	barren	NCAPH	0.66
BKN21481	CG3329	FBgn0023174	Proteasome beta2 subunit	PSMB10, PSMB7	0.67
BKN29219	CG3455	FBgn0028685	Regulatory particle triple-A ATPase 4	WDR12, PSMC6, PSMC5, PSMC2	0.67
BKN25523	CG18471	FBgn0024232	gprs	ENC1, KLHL25	0.67
BKN21943	CG9104	FBgn0030800	Nitrogen permease regulator-like 2	NPRL2, ZMYND10	0.67
BKN29413	CG9878	FBgn0027360	Translocase of inner membrane 10	TIMM10	0.67
BKN22741	CG3180	FBgn0003276	RNA polymerase II 140kD subunit	POLR3B, POLR2B	0.67
BKN27806	CG14791	FBgn0025382	Rab27	RAB27B, RAB27A	0.67
BKN30297	CG14564	FBgn0037131	-	-	0.67
BKN31650	CG11278	FBgn0036341	Syntaxin 13	STX17, TSNARE1, STX7, STX12	0.67
BKN26740	CG13656	FBgn0039307	-	-	0.67
BKN20730	CG1528	FBgn0028968	Coat Protein (coatomer) gamma	COPG2, COPG1	0.67
BKN45278	CG14463	FBgn0037535	Phosphatidylinositol glycan anchor biosynthesis class	PIGH	0.67
BKN25958	CG30073	FBgn0050073	Odorant-binding protein 50b	-	0.67
BKN32626	CG6689	FBgn0037877	-	ZNF296, ZNF691, ZNF560, KLF17, WT1, I	0.67
BKN45442	CG15151	FBgn0243486	reduced ocelli	PPP1R42	0.67
BKN29232	CG17737	FBgn0035423	eukaryotic translation initiation factor 1	EIF1B, EIF1	0.67
BKN20315	CG3820	FBgn0010660	Nucleoporin 214kD	NUP214	0.67
BKN41326	CG33838	FBgn0053838	His2A:CG33838	HIST2H2AB, H2AFB2, H2AFY, HIST1H2A	0.67
BKN22248	CG3587	FBgn0023521	-	KTI12	0.67
BKN21483	CG4931	FBgn0038320	specifically Rac1-associated protein 1	CYFIP2, CYFIP1	0.67
BKN45209	CG3068	FBgn0000147	aurora A	AURKC, AURKB, AURKA	0.67
BKN20264	CG7115	FBgn0027515	-	ILKAP, PPM1L, PPM1K, PPM1E, PP2D1,	0.67
BKN26474	CG17525	FBgn0063496	Glutathione S transferase E4	GSTT2, EEF1G, GSTT1, GSTT2B, MARS,	0.67
BKN45322	CG12653	FBgn0000233	buttonhead	EGR1, ZNF526, SP7, SP8, EGR3, SP9, SI	0.67
BKN26505	CG15208	FBgn0030247	-	C21orf2	0.67
BKN25475	CG7574	FBgn0026263	bip1	-	0.67
BKN42189	CG2512	FBgn0003885	alpha-Tubulin at 84D	TUBA4A, TUBA1B, TUBA3D, TUBA1C, TU	0.67
BKN42655	CR34643	FBgn0082966	snoRNA:Psi28S-3327b	-	0.67
BKN30929	CG11401	FBgn0037170	thioredoxin reductase 2	TXNRD1, TXNRD2, GSR, TXNRD3	0.67
BKN31574	CG11562	FBgn0031247	-	PET117	0.67
BKN26890	CG13309	FBgn0035933	-	-	0.67
BKN21403	CG4274	FBgn0001086	fizzy	CDC20B, FZR1, CDC20	0.67
BKN24936	CG13826	FBgn0040590	cap-n-collar	BACH1, NFE2L1, KLHL6, NFE2, NFE2L2,	0.68
BKN25201	CG4481	FBgn0028431	Glutamate receptor IB	GRIK2, GRIK3, GRIA3, GRIA1, GRIK1, GI	0.68
BKN22895	CG8580	FBgn0082598	akirin	AKIRIN1, AKIRIN2	0.68
BKN20602	CG1810	FBgn0030556	mRNA-capping-enzyme	RNGTT, DUSP11	0.68
BKN26856	CG31513	FBgn0051513	-	-	0.68
BKN21756	CG18616	FBgn0260444	CCR4-NOT transcription complex subunit 10	CNOT10	0.68
BKN28526	CG3931	FBgn0034879	Rrp4	EXOSC2	0.68
BKN25639	CG1618	FBgn0000346	comatose	NSF	0.68
BKN45200	CG4088	FBgn0005654	Origin recognition complex subunit 3	ORC3	0.68
BKN24935	CG4319	FBgn0011706	reaper	TSC1, RIN1	0.68
BKN41583	CG31449	FBgn0013277	Heat-shock-protein-70Ba	NCKAP1, HSPA6, HSPA1B, HSPA5, NCK	0.68
BKN41499	CG33821	FBgn0053821	His3:CG33821	HIST1H3J, HIST2H3C, HIST1H3F, HIST1I	0.68
BKN26584	CG16753	FBgn0035393	-	FAM207A	0.68
BKN29819	CG1115	FBgn0037299	Vacuolar protein sorting 37B	VPS37C, VPS37B, C9orf85, VPS37A, VPS	0.68
BKN27317	CG7581	FBgn0025457	Bub3	BUB3	0.68
BKN23027	CG2009	FBgn0026262	bip2	ING4, TAF8, ING2, ING3, ING1, ING5, TAI	0.68
BKN33369	CG11362	FBgn0034737	-	-	0.68
BKN31497	CG3448	FBgn0035996	-	-	0.68

BKN42114	CG10679	FBgn0032725	Nedd8	RAD23B, UBA52, UBB, UBD, NEDD8, ISG	0.68
BKN20853	CG1471	FBgn0039774	Ceramidase	ASAH2, ASAH2B	0.68
BKN29269	CG11525	FBgn0039858	Cyclin G	CCNB2, CCNG1, CCNB3, CCNI2, CCNB1	0.68
BKN33780	HDC04339	FBgn0250870	-	-	0.69
BKN45817	CG6061	FBgn0033846	Myb-interacting protein 120	LIN54, TESMIN	0.69
BKN28188	CG3630	FBgn0023540	-	ABRA, ABRACL	0.69
BKN29170	CG1796	FBgn0030365	Transport and Golgi organization 4	PLRG1	0.69
BKN40722	CG34042	FBgn0065110	pickpocket 10	SCNN1G, ASIC3, ASIC1, ASIC4, SCNN1E	0.69
BKN42380	CG42314	FBgn0004865	plasma membrane calcium ATPase	ATP2B1, ATP2B3, ATP2B2, ATP2B4	0.69
BKN32589	CG14521	FBgn0039617	Dpr-interacting protein gamma	JAM2, ESAM, NTM, VSIG1, GPA33, CXAD	0.69
BKN21370	CG3416	FBgn0002787	Regulatory particle non-ATPase 8	PSMD7	0.69
BKN20820	CG1972	FBgn0039691	Integrator 11	CPSF3, CPSF3L	0.69
BKN21112	CG8598	FBgn0035766	Establishment of cohesion	POLI, REV1, POLH, POLK, ESCO2, ESCO	0.69
BKN31810	CG12115	FBgn0030097	-	-	0.69
BKN20843	CG8975	FBgn0011704	Ribonucleoside diphosphate reductase small subunit	RRM2, RRM2B	0.69
BKN20923	CG1877	FBgn0015509	Cullin 1	CUL1, CACUL1	0.69
BKN45758	CG1772	FBgn0010316	dacapo	CDKN1B, CDKN1A, CDKN1C	0.69
BKN46099	CG9032	FBgn0014391	stunted	ATP5E	0.69
BKN20346	CG6699	FBgn0025724	Coat Protein (coatomer) beta'	COPA, COPB2	0.69
BKN21769	CG11103	FBgn0030522	-	TM2D2	0.69
BKN25372	CG30052	FBgn0050052	Odorant-binding protein 49a	-	0.69
BKN45039	CG8560	FBgn0035781	-	CPA4, CPA3, CPA5, CPA2, CPB1, CPA1,	0.69
BKN33804	HDC08545	FBgn0054047	-	-	0.69
BKN29271	CG8922	FBgn0002590	Ribosomal protein S5a	MRPS7, RPS5	0.69
BKN21518	CG4332	FBgn0030456	-	CLPTM1, CLPTM1L	0.69
BKN21755	CG11228	FBgn0034453	hippo	STK4, STK3	0.69
BKN27999	CG10671	FBgn0035586	Fat storage inducing transmembrane protein	FITM1, FITM2	0.69
BKN41228	CG33898	FBgn0053898	His2B:CG33898	HIST2H2BF, HIST1H2BB, HIST1H2BC, HI	0.69
BKN24419	CG4784	FBgn0036619	Cuticular protein 72Ec	-	0.69
BKN24628	CG15877	FBgn0035337	-	C7orf50	0.70
BKN24021	CG4877	FBgn0036624	RING-associated factor 2	ZMYND10	0.70
BKN24254	CG33227	FBgn0085399	-	-	0.70
BKN28595	CG4747	FBgn0043456	-	HDGFRP2, GLYR1, PSIP1, HIBADH	0.70
BKN24984	CG15541	FBgn0085462	-	-	0.70
BKN42505	CG42330	FBgn0259232	Down syndrome cell adhesion molecule 4	NRCAM, MYLK4, SPEG, NFASC, DSCAM	0.70
BKN46515	CG3756	FBgn0031657	-	POLR1C	0.70
BKN22357	CG4141	FBgn0015279	Pi3K92E	PIK3CB, PIK3CD, PIK3CA, PIK3CG	0.70
BKN26990	CG4875	FBgn0030801	Receptor component protein	POLR2D, CRCP, ASL	0.70
BKN21636	CG2252	FBgn0004656	female sterile (1) homeotic	BRDT, BAZ1A, BAZ2B, CECR2, BRD4, C	0.70
BKN32873	CG3493	FBgn0034854	Golgin-245	GOLGA4, GOLGB1, CENPE, CENPF	0.70

475 dsRNAs (467 genes) that decrease dPum activity

BKN#	CG#	Flybase_ID	gene name	human orthologs	FF-PRE/Ren ratio
BKN25067	CG30053	FBgn0050053	-	-	2.75
BKN40434	CG1957	FBgn0027873	Cleavage and polyadenylation specificity factor 100	CPSF2	2.51
BKN30861	CG13715	FBgn0035564	axotactin	SPINT1, CNTNAP4, CNTNAP2, CNTNAP1	2.44
BKN20249	CG18497	FBgn0016977	split ends	RBM15B, RBM15, SPEN	2.38
BKN29156	CG7467	FBgn0003013	osa	ARID3C, ARID3A, ARID1A, ARID4A, ARID	2.37
BKN27450	CG7697	FBgn0027841	Cleavage stimulation factor 64 kD subunit	CSTF2T, CSTF2	2.26
BKN20232	CG4699	FBgn0038364	non-specific lethal 1	KANSL1L, KANSL1	2.09
BKN21271	CG5684	FBgn0036239	Pop2	CNOT7, CNOT8	1.98
BKN27909	CG7055	FBgn0030093	Brahma associated protein 111kD	SMARCE1	1.98
BKN27752	CG15112	FBgn0000578	enabled	VASP, ENAH, EVL, SPRED3	1.97
BKN32717	CG5442	FBgn0040286	SR family splicing factor SC35	SRSF12, SRSF2, RBMXL1, SRSF8, RBM	1.97
BKN32049	CG18371	FBgn0033893	-	ACYP1, ACYP2	1.91
BKN32690	CG10107	FBgn0035713	veloren	SENP6, SENP7, TLR8, CD180, TLR4	1.91
BKN31781	CG18290	FBgn0000046	Actin 87E	ACTL7A, POTEI, ACTR10, ACTL10, ACTF	1.90
BKN25485	CG2052	FBgn0039905	datilografo	ZBTB48, ZXDB, ZNF362, ZXDA, ZXDC	1.88
BKN34554	HDC14090		-	-	1.86
BKN31691	CG2368	FBgn0004399	pipsqueak	BACH2	1.86

BKN42338	CG40392	FBgn0058392	-	-	1.85
BKN31630	CG15867	FBgn0085427	-	-	1.84
BKN20344	CG5942	FBgn0000212	brahma	SMARCA4, SMARCA2, HELLS	1.84
BKN22809	CG3242	FBgn0004892	sister of odd and bowl	ZBTB2, GFI1, OSR1, OSR2	1.79
BKN31250	CG17343	FBgn0032751	-	-	1.78
BKN24343	CG31714	FBgn0032180	-	ADGRB3	1.78
BKN23222	CG14796	FBgn0025390	Mucin related 2B	MUC16, MUC17, MUC5B, SSPO, MUC4, I	1.78
BKN32776	CG33261	FBgn0013263	Trithorax-like	NACC2, ZBTB44, BCL6B, ZBTB46, ZBTB3	1.77
BKN20604	CG9005	FBgn0033638	-	FAM214A, FAM214B	1.76
BKN31738	CG5442	FBgn0040286	SR family splicing factor SC35	SRSF12, SRSF2, RBMXL1, SRSF8, RBMY	1.73
BKN33865	HDC15754		-	-	1.73
BKN41059	CG40121	FBgn0058121	-	-	1.73
BKN23928	CG6765	FBgn0035903	-	ZBTB9	1.73
BKN29161	CG4027	FBgn0000042	Actin 5C	POTEI, ACTR1A, POTEB2, ACTG1, ACTL	1.72
BKN25474	CG33230	FBgn0053230	-	SETD6, SETD3, SETD4	1.71
BKN29594	CG3056	FBgn0024987	sister-of-Sex-lethal	ELAVL1, ELAVL2, ELAVL3, ELAVL4	1.70
BKN23674	CG5815	FBgn0027574	-	PLD2, SPTY2D1	1.70
BKN33868	HDC16553		-	-	1.70
BKN45595	CG7697	FBgn0027841	Cleavage stimulation factor 64 kD subunit	CSTF2T, CSTF2	1.69
BKN22697	CG33175	FBgn0085443	sprint	RIN3, RIN2, RIN1, RINL	1.69
BKN23588	CG18178	FBgn0036035	-	MAK16	1.69
BKN32872	CG30176	FBgn0034918	Partner of Y14 and Mago	PYM1	1.69
BKN21154	CG7052	FBgn0041182	Thioester-containing protein 2	CD109, C4A, C4B_2, A2M, C5, A2ML1, C3	1.69
BKN21312	CG8455	FBgn0031997	Post-GPI attachment to proteins 5	MPPE1	1.68
BKN33387	HDC09424		-	-	1.67
BKN33123	CG15353	FBgn0040718	-	-	1.67
BKN20229	CG32677	FBgn0052677	X11Lbeta	APBA3, APBA2, APBA1	1.66
BKN45606	CG10107	FBgn0035713	veloren	SENP6, SENP7, TLR8, CD180, TLR4	1.66
BKN31641	CG11742	FBgn0037594	Odorant receptor 85d	-	1.66
BKN26243	CG9792	FBgn0041711	yellow-e	DPM3	1.65
BKN46391	CG12051	FBgn0000043	Actin 42A	ACTL8, ACTRT3, POTEH, ACTR10, ACTL	1.64
BKN31268	CG7334	FBgn0036191	Sugar baby	SCN10A, MFSD6, PTGS2, MFSD6L	1.63
BKN21288	CG2097	FBgn0037371	Symplekin	SYMPK	1.62
BKN32180	CG8420	FBgn0037664	-	-	1.62
BKN21059	CG5899	FBgn0032157	Etl1	SMARCAD1	1.61
BKN21910	CG4303	FBgn0025463	Brahma associated protein 60kD	SMARCD1, ASIC1, SMARCD3, SMARCD2	1.61
BKN32909	CG32077	FBgn0014368	no optic lobe	-	1.61
BKN20241	CG18811	FBgn0042134	Caprin	C1QL1, C1QL4, AMACR, CBLN3, CAPRIN	1.60
BKN23252	CG3044	FBgn0029913	Chitinase 11	CHI3L2, OVGP1, CHIT1, CHIA, SRL, CHI3	1.60
BKN33041	CG12621	FBgn0032627	beat-IIIa	CADM2	1.60
BKN33106	CG8396	FBgn0015299	Single stranded-binding protein c31A	SUB1	1.59
BKN32786	CG32404	FBgn0052404	Cuticular protein 65Aw	-	1.59
BKN34662	HDC19986		-	-	1.59
BKN22295	CG2171	FBgn0086355	Triose phosphate isomerase	TPI1	1.59
BKN41659	CG34167	FBgn0085196	-	KRTAP5-6	1.58
BKN29113	CG1983	FBgn0039751	-	PROSC	1.58
BKN20534	CG7698	FBgn0038636	Cleavage and polyadenylation specificity factor 73	CPSF3L, CPSF3	1.58
BKN46312	CG16772	FBgn0032835	-	-	1.58
BKN22098	CG7144	FBgn0025687	Lysine ketoglutarate reductase/saccharopine dehydro	AASS	1.57
BKN32796	CG31629	FBgn0085407	PDGF- and VEGF-related factor 3	-	1.57
BKN20794	CG10653	FBgn0001202	hook	HOOK3, HOOK1, HOOK2	1.57
BKN34645	HDC14458		-	-	1.56
BKN33839	HDC14293		-	-	1.56
BKN22341	CG6181	FBgn0032340	Ge-1	EDC4	1.56
BKN20265	CG3634	FBgn0037026	-	ST7L, ST7	1.55
BKN20582	CG15014	FBgn0035532	-	THUMPD1	1.55
BKN25664	CG31826	FBgn0051826	-	RLBP1, CLVS1, CLVS2, TTPAL, SEC14L1	1.54
BKN27684	CG10967	FBgn0010715	Autophagy-related 1	ULK2, ULK3, PDIK1L, STK35, ULK1	1.54
BKN22201	CG6084	FBgn0086254	-	AKR1C3, AKR1C4, AKR1A1, AKR1D1, AK	1.54
BKN32935	CG4312	FBgn0002869	Metallothionein B	-	1.54
BKN30471	CG1143	FBgn0035359	-	-	1.54
BKN34457	HDC10902		-	-	1.54

BKN29340	CG7736	FBgn0037084	Syntaxin 6	STX6, STX8, STX10	1.54
BKN22318	CG10078	FBgn0041194	Phosphoribosylamidotransferase 2	PPAT	1.53
BKN40127	CG33671	FBgn0061359	-	MVK	1.53
BKN20850	CG10542	FBgn0086694	Bre1	RNF20, PEX10, TRIM39, RNF5, RNF185,	1.53
BKN29389	CG6153	FBgn0032445	-	PITHD1	1.53
BKN24862	CG4399	FBgn0010110	enhanced adult sensory threshold	-	1.53
BKN29721	CG14636	FBgn0037217	-	-	1.52
BKN20230	CG17018	FBgn0039972	-	KIAA0430	1.52
BKN24621	CG10124	FBgn0035709	eukaryotic translation initiation factor 4E4	SESN3, EIF4E1B, SESN1, SESN2, EIF4E	1.51
BKN26032	CG5409	FBgn0011743	Actin-related protein 53D	POTEJ, ACTB, POTEE, ACTRT1, ACTR8,	1.51
BKN22845	CG8734	FBgn0033315	Galactosyltransferase II	B3GALT6, B3GALNT2	1.51
BKN28039	CG1467	FBgn0031106	Syntaxin 16	STX7, STX16, STX12	1.51
BKN31533	CG30354	FBgn0050354	Ubiquinol-cytochrome c reductase 11 kDa subunit-like	UQCRH, UQCRHL	1.51
BKN27774	CG7062	FBgn0015793	Rab19	RAB33B, RAB12, RABL6, RAB1B, RAB19	1.51
BKN30809	CG3119	FBgn0031466	-	C1GALT1, C1GALT1C1	1.50
BKN29150	CG9220	FBgn0030662	-	CHSY3, CSGALNACT2, CSGALNACT1, C	1.50
BKN22352	CG4376	FBgn0000667	alpha actinin	SPTAN1, SPTBN1, SPTBN2, ACTN3, GAS	1.50
BKN45210	CG17122	FBgn0036962	-	CFAP157	1.50
BKN42426	CG12997	FBgn0030831	-	-	1.49
BKN22638	CG6510	FBgn0010409	Ribosomal protein L18A	RPL18A	1.49
BKN22374	CG10011	FBgn0039590	-	CTTNBP2, ANKRD50	1.49
BKN45137	CG5874	FBgn0038872	Negative elongation factor A	NELFA	1.49
BKN25256	CG15445	FBgn0031161	-	NUB1	1.49
BKN29402	CG10652	FBgn0086710	Ribosomal protein L30	RPL30	1.49
BKN22515	CG32133	FBgn0052133	PAX transcription activation domain interacting protei	MDC1, PAXIP1	1.49
BKN26785	CG10183	FBgn0039093	-	-	1.48
BKN34530	HDC09489		-	-	1.48
BKN34258	HDC08564		-	-	1.48
BKN41528	CR34623	FBgn0082957	snoRNA:Psi28S-3405d	-	1.48
BKN40461	CG42236	FBgn0250861	Ran-binding protein M	SPRYD3, RANBP10, RANBP9	1.48
BKN42498	CG33774	FBgn0053774	-	OST4	1.48
BKN29268	CG8529	FBgn0033739	Dystrobrevin	DTNA, TUSC1, DTNB, DRP2	1.48
BKN40384	CG41603	FBgn0085719	-	-	1.47
BKN27682	CG4867	FBgn0040239	bc10	BLCAP	1.47
BKN24746	CG13310	FBgn0035928	-	-	1.46
BKN28569	CG3412	FBgn0023423	supernumerary limbs	FBXW7, DAW1, WDR64, BTRC, FBXW11	1.46
BKN33080	CG2941	FBgn0029686	-	-	1.46
BKN22983	CG10161	FBgn0040227	eukaryotic translation initiation factor 3 subunit d1	EIF3D	1.46
BKN21266	CG11025	FBgn0028372	isopeptidase-T-3	UBAC1	1.46
BKN28958	CG18214	FBgn0024277	trio	SESTD1, MCF2, ARHGEF25, PLEKHG4B	1.45
BKN22414	CG4698	FBgn0010453	Wnt oncogene analog 4	WNT6, WNT10A, WNT9B, WNT8A, WNT3	1.45
BKN29931	CG1322	FBgn0004606	Zn finger homeodomain 1	ZEB1, MECOM, ZNF853, ZNF91, ZNF236	1.45
BKN20215	CG7861	FBgn0033055	Tubulin-specific chaperone E	TBCE, TBCEL	1.45
BKN27824	CG5020	FBgn0020503	Cytoplasmic linker protein 190	NBPF8, NBPF1, NBPF3, MMRN2, CCDC2	1.45
BKN23056	CG6439	FBgn0038922	Isocitrate dehydrogenase 3b	IDH3A, IDH3B, IDH3G	1.45
BKN27884	CG2006	FBgn0039664	-	DTWD1	1.45
BKN29394	CG1975	FBgn0028408	DNA fragmentation factor-related protein 2	CIDEA, CIDEC, DFFA, CIDEB	1.45
BKN20278	CG15270	FBgn0028879	-	ANO5, ANO10, ANO8, ANO4, ANO7, ANC	1.44
BKN40323	CR33221	FBgn0053221	-	-	1.44
BKN28294	CG10603	FBgn0032720	mitochondrial ribosomal protein L13	MRPL13	1.44
BKN24250	CG31224	FBgn0051224	-	FIZ1, ZBTB44, ZNF579, ZNF618	1.44
BKN29052	CG7807	FBgn0023417	Transcription factor AP-2	TFAP2C, TFAP2D, TFAP2E, TFAP2B, TF/	1.44
BKN32894	CG10910	FBgn0034289	-	CDA, MAGED4, MAGED2, MAGED1, MAC	1.44
BKN27861	CG11166	FBgn0033166	ELL-associated factor	EAF1, EAF2	1.43
BKN21775	CG9854	FBgn0015949	hiiragi	PAPOLA, PAPOLG, PAPOLB	1.43
BKN30397	CG1648	FBgn0033446	-	PLIN3, PLIN1, PLIN5, ADIRF, PLIN2	1.43
BKN40206	CR42954	FBgn0046818	mir-6-2 stem loop	-	1.42
BKN40307	CR32618	FBgn0011981	transfer RNA:Serine-AGA 3-2	-	1.42
BKN45785	CG43225	FBgn0035564	axotactin	SPINT1, CNTNAP4, CNTNAP2, CNTNAP1	1.42
BKN27766	CG9921	FBgn0030743	-	HIGD2A, HIGD1B, HIGD1A, HIGD1C	1.42
BKN32968	CG15470	FBgn0029731	-	KCNK2, KCNK16, KCNK7, KCNK5, KCNK	1.42
BKN33728	HDC05690		-	-	1.42

BKN22300	CG9915	FBgn0030738	-	IWS1	1.42
BKN21305	CG32705	FBgn0085479	-	-	1.41
BKN33070	CG15461	FBgn0040649	-	-	1.41
BKN42285	CG6783	FBgn0037913	fatty acid binding protein	RBP2, FABP4, FABP1, RBP5, FABP5, FA	1.41
BKN28859	CG7007	FBgn0028662	Vacuolar H[+] ATPase PPA1 subunit 1	ATP6V0B	1.41
BKN42530	CG34176	FBgn0085205	-	-	1.41
BKN23191	CG15390	FBgn0031419	-	MTERF4	1.41
BKN20592	CG17256	FBgn0029970	Nek2	NEK7, NEK4, NEK2, NEK11, NEK8, STKL	1.41
BKN29048	CG9755	FBgn0003165	pumilio	PUM1, PUM2	1.41
BKN34012	HDC02762		-	-	1.41
BKN30191	CG10571	FBgn0015904	araucan	IRX6, MEIS1, TGIF2, IRX4, IRX2, IRX5, T	1.41
BKN42552	CG18160	FBgn0039980	-	-	1.41
BKN30894	CG15279	FBgn0028886	-	SLC6A5, SLC6A9, SLC6A17, SLC6A20, S	1.40
BKN32994	CG10843	FBgn0033397	Cyp4p3	CYP4A22, CYP4A11, CYP20A1, CYP4F1	1.40
BKN32014	CG17622	FBgn0038998	wide awake	ANKFN1	1.40
BKN20290	CG31937	FBgn0031360	-	HSD11B1L, RDH8, ELOVL7, HSD17B1, D	1.40
BKN22416	CG7050	FBgn0038975	Neurexin 1	NRXN2, NRXN3, NRXN1	1.40
BKN27772	CG2812	FBgn0034931	-	WDR47	1.40
BKN33107	CG5539	FBgn0034907	-	REEP3, REEP4, REEP2, REEP1	1.40
BKN22692	CG16947	FBgn0031816	-	RCHY1	1.40
BKN23716	CG10217	FBgn0039113	-	-	1.40
BKN21664	CG7471	FBgn0015805	Histone deacetylase 1	HDAC1, HDAC3, HDAC8, HDAC2	1.40
BKN31779	CG12479	FBgn0040871	-	MINOS1, MINOS1-NBL1	1.40
BKN28625	CG10233	FBgn0087005	retinophilin	MORN1, JPH1, MORN3, RSPH10B2, RSP	1.40
BKN45970	CG2903	FBgn0031450	Hepatocyte growth factor regulated tyrosine kinase su	ZFYVE19, GGA1, GGA3, TOM1L2, ZFYVE	1.40
BKN20292	CG1848	FBgn0041203	LIM domain kinase 1	MAP3K21, LIMK2, LRRK1, TESK2, LIMK1	1.39
BKN42402	CG41325	FBgn0085615	-	-	1.39
BKN34390	HDC19711		-	-	1.39
BKN30630	CG5738	FBgn0022238	lola like	ZBTB43, ZBTB9, ZBTB37, ZBTB12	1.39
BKN28710	CG2099	FBgn0037328	Ribosomal protein L35A	RPL35A	1.39
BKN32480	CG9656	FBgn0001138	grain	GATA5, TRPS1, ZGLP1, GATA1, GATA6,	1.39
BKN33928	HDC10263		-	-	1.39
BKN30046	CG13011	FBgn0030771	singles bar	CMTM2, CMTM1, CMTM7, CMTM3, CKLF	1.39
BKN45374	CG10177	FBgn0039083	-	DCLK1, DAPK2, OBSCN, DAPK1, MYLK3	1.39
BKN28734	CG3195	FBgn0034968	Ribosomal protein L12	RPL12	1.39
BKN41904	CG41481	FBgn0084041	-	-	1.39
BKN28711	CG1475	FBgn0037351	Ribosomal protein L13A	RPL13A	1.39
BKN26278	CG3331	FBgn0000527	ebony	AASDH	1.39
BKN34006	HDC02763		-	-	1.38
BKN24775	CG15780	FBgn0250904	-	MYL1, MYL6, MYL6B, MYL4, MYL3	1.38
BKN32575	CG5596	FBgn0002772	Myosin alkali light chain 1	MYL6, MYL4, MYL6B, MYL1, MYL3	1.38
BKN22604	CG18735	FBgn0042098	-	PRSS1, CELA3A, TMPRSS4, PRSS3, ST1	1.38
BKN34076	HDC02764	FBgn0085196	-	-	1.38
BKN32676	CG14358	FBgn0038199	CCHamide-1	-	1.38
BKN27102	CG15765	FBgn0029814	-	LINC00083	1.38
BKN28468	CG7018	FBgn0005658	Ets at 65A	ERF, ETS2, GABPA, ELK4, ETV4, ETS1, E	1.37
BKN32013	CR33749	FBgn0065104	small non-messenger RNA 158	-	1.37
BKN26287	CG14853	FBgn0038246	-	ERICH2	1.37
BKN40951	CR32287	FBgn0052287	transfer RNA:Cysteine-GCA 1-3	-	1.37
BKN26394	CG6792	FBgn0032401	Promyelocytic leukemia zinc finger	ZBTB38, ZBTB42, ZBTB14, ZBTB2, ZBTB	1.37
BKN45892	CG11236	FBgn0031860	-	DDO, DAO, METTL24	1.37
BKN28748	CG6563	FBgn0038306	Arginine methyltransferase 3	PRMT2, ETFBKMT, NDUFAF5, PRMT3, P	1.37
BKN24908	CG8290	FBgn0026573	ADD domain-containing protein 1	DNMT3A, DNMT3L, ATRX, DNMT3B	1.37
BKN31245	CG4220	FBgn0004858	elbow B	ZNF703, ZNF503	1.37
BKN28599	CG12054	FBgn0039831	-	JAZF1	1.37
BKN25902	CG16940	FBgn0035111	Dis3 like 3'-5' exoribonuclease 2	DIS3L2, HELZ2, DIS3, DIS3L	1.37
BKN24602	CG15889	FBgn0038128	Ravus	-	1.37
BKN33711	HDC02932	FBgn0032631	-	-	1.37
BKN32738	CG32240	FBgn0052240	-	-	1.37
BKN45854	CG8310	FBgn0040377	Vacuolar H[+] ATPase 36kD subunit 3	ATP6V1D	1.37
BKN31226	CG13947	FBgn0031277	-	-	1.37
BKN28383	CG8639	FBgn0033313	Calcium-independent receptor for alpha-latrotoxin	ADGRL1, ADGRF4, ADGRE3, EVA1C, CF	1.37

BKN28712	CG3717	FBgn0013432	bcn92	LYRM4	1.37
BKN27944	CG5676	FBgn0032200	-	FUNDC1, FUNDC2	1.37
BKN40293	CG43443	FBgn0085226	hu li tai shao	ADD3, ADD2, ADD1	1.36
BKN22471	CG7793	FBgn0001965	Son of sevenless	RASGRF2, RAPGEF1, RASGRP2, RASGI	1.36
BKN24414	CG17453	FBgn0033982	Cyp317a1	CYP3A43, TBXAS1, PTGIS, CYP3A5, CYI	1.36
BKN31419	CG4710	FBgn0016926	Pinocchio	-	1.36
BKN26752	CG30170	FBgn0004581	benign gonial cell neoplasm	DHX37, DHX30, TDRD9, DHX9, DHX57, Y	1.36
BKN28210	CG3582	FBgn0017457	U2 small nuclear riboprotein auxiliary factor 38	ZRSR2, U2AF1L4, U2AF1, U2AF1L5	1.36
BKN34351	HDC09245		-	-	1.36
BKN31981	CG16791	FBgn0038881	-	-	1.36
BKN45857	CG32240	FBgn0052240	-	-	1.36
BKN20245	CG8266	FBgn0033339	Secretory 31	SEC31A, SEC31B	1.36
BKN28708	CG7048	FBgn0038976	Prefoldin 5	PFDN5	1.36
BKN33623	HDC15759		-	-	1.36
BKN21653	CG1064	FBgn0011715	Snf5-related 1	SMARCB1	1.36
BKN40036	CG17665	FBgn0039997	Integrator 3	INTS3, NPR1, NPR2	1.35
BKN29392	CG4205	FBgn0011769	Ferredoxin 1	FDX1, FDX1L, COX15	1.35
BKN22598	CG2746	FBgn0002607	Ribosomal protein L19	RPL19	1.35
BKN45672	CG33485	FBgn0038282	defective proboscis extension response 9	-	1.35
BKN40695	CG5172	FBgn0030830	-	-	1.35
BKN25648	CG11280	FBgn0010452	tartan	BGN, LRRC70, LRRN3, CPN2, LRFN1, LI	1.35
BKN32844	CG13578	FBgn0035000	-	-	1.35
BKN32752	CG10205	FBgn0033970	-	-	1.35
BKN25154	CG14317	FBgn0038566	-	-	1.35
BKN32537	CG3361	FBgn0039507	martik	-	1.34
BKN21326	CG1818	FBgn0033428	Updo	UROD	1.34
BKN24535	CG31975	FBgn0051975	-	-	1.34
BKN20340	CG10354	FBgn0031868	Rat1 5'-3' exoribonuclease	XRN1, XRN2	1.34
BKN22447	CG9207	FBgn0031873	Gas41	MLLT3, YEATS2, MLLT1, YEATS4	1.34
BKN32793	CG12384	FBgn0033624	-	DAPL1, DAP	1.34
BKN42580	CG18773	FBgn0020643	Lcp65Ab2	-	1.34
BKN45919	CG3708	FBgn0040345	-	NAP1L4, NAP1L2, NAP1L5, NAP1L1, NAI	1.34
BKN28740	CG17252	FBgn0026149	BCL7-like	BCL7B, BCL7C, BCL7A	1.34
BKN34016	HDC02979		-	-	1.34
BKN30769	CG13252	FBgn0037016	-	OTOGL, VWCE, NELL2, NELL1, FRAS1, (	1.34
BKN28452	CG2014	FBgn0039669	NADH dehydrogenase (ubiquinone) 20 kDa subunit-li	NDUFS7	1.34
BKN22501	CG7456	FBgn0032258	-	COG4	1.34
BKN28706	CG4254	FBgn0011726	twinstar	CFL1, CFL2, DSTN	1.34
BKN29229	CG10751	FBgn0024196	roadblock	DYNLRB1, DYNLRB2	1.34
BKN40612	CG11919	FBgn0033564	Peroxin 6	PEX6	1.33
BKN29437	CG9578	FBgn0031094	-	TIPRL	1.33
BKN22290	CG31753	FBgn0045852	hamlet	ZNF85, ZNF605, ZNF808, ZFP62, ZNF721	1.33
BKN29849	CG14035	FBgn0031685	Muscle-specific protein 300 kDa	SMTNL2, SYNE2, MACF1, EVPLL, SMTNI	1.33
BKN29393	CG11654	FBgn0014455	Adenosylhomocysteinase	AHCYL1, AHCY, AHCYL2	1.33
BKN33083	CG5630	FBgn0038842	hadley	-	1.33
BKN31818	CG15286	FBgn0028531	-	KCMF1	1.33
BKN33120	CG15489	FBgn0085424	nubbin	POU6F2, POU3F1, POU1F1, POU5F2, PC	1.33
BKN42277	CG33242	FBgn0053242	Ste:CG33242	CSNK2B	1.33
BKN28784	CG4921	FBgn0016701	Rab4	RAB2A, MIA, RAB14, EGLN2, RAB4A, RA	1.33
BKN45347	CG5942	FBgn0000212	brahma	SMARCA4, SMARCA2, HELLS	1.33
BKN32129	CG13594	FBgn0035041	-	-	1.33
BKN41139	CG9369	FBgn0002577	miniature	-	1.33
BKN40319	CG33718	FBgn0044419	Pmi	TMEM11	1.33
BKN20860	CG8233	FBgn0033897	Reduction in Cnn dots 1	KANSL3	1.33
BKN41706	CG41312	FBgn0085608	-	-	1.33
BKN27320	CG5445	FBgn0030838	-	NBR1, C6orf106	1.33
BKN40660	CG33251	FBgn0053251	-	-	1.33
BKN23731	CG10192	FBgn0260634	eukaryotic translation initiation factor 4G2	EIF4G1, EIF4G2, EIF4G3	1.33
BKN25688	CG15179	FBgn0037462	sungrazer	CIB1, CHP2, CIB4, EFCAB1, TESC, PPP3	1.33
BKN21075	CG17369	FBgn0005671	Vacuolar H[+]-ATPase 55kD subunit	ATP6V1B2, ATP6V1B1	1.32
BKN33108	CG17086	FBgn0032310	SET and MYND domain containing, arthropod-specifi	SMYD4, ERLIN1, SMYD5, SMYD3, SMYD	1.32
BKN40074	CR40573	FBgn0085748	-	-	1.32

BKN33172	CG14063	FBgn0040618	-	-	1.32
BKN45165	CG3350	FBgn0039509	bigmax	MLX	1.32
BKN21682	CG12121	FBgn0030109	-	GPR107, GPR108	1.32
BKN24651	CG9668	FBgn0003250	Rhodopsin 4	RGR, OPN1MW3, OPN3, RRH, OPN4, RH	1.32
BKN31559	CG31206	FBgn0051206	-	-	1.32
BKN33235	CG18336	FBgn0040763	-	FAM166B, FAM166A	1.32
BKN28864	CG8186	FBgn0022097	Vacuolar H[+] ATPase 36kD subunit 1	ATP6V1D	1.32
BKN23235	CG32790	FBgn0052790	-	ST18, MYT1L, MYT1	1.32
BKN29239	CG14213	FBgn0031047	Required for cell differentiation 1	CNOT9	1.32
BKN21289	CG4039	FBgn0025815	Minichromosome maintenance 6	MCM6, MCM8, MCMDC2	1.32
BKN30826	CG31699	FBgn0051699	-	-	1.32
BKN28208	CG2972	FBgn0030177	-	NOB1	1.32
BKN28772	CG7041	FBgn0030082	Heterochromatin Protein 1b	CBX3, C15orf57, CDY1, CDY2A, CDY1B,	1.32
BKN21416	CG6046	FBgn0024491	Bicoid interacting protein 1	SAP18	1.31
BKN21008	CG11396	FBgn0037022	-	TRAPPC12	1.31
BKN34089	HDC05888		-	-	1.31
BKN27811	CG33096	FBgn0053096	-	ABHD13, ABHD12, ABHD17B, ABHD17A,	1.31
BKN28204	CG11137	FBgn0037199	-	EMC4	1.31
BKN24777	CG8029	FBgn0033393	Vacuolar H[+] ATPase AC45 accessory subunit	ATP6AP1, ATP6AP1L	1.31
BKN34516	HDC08154		-	-	1.30
BKN27800	CG6030	FBgn0016120	ATP synthase, subunit D	ATP5H	1.30
BKN28127	CG32343	FBgn0052343	Ada2a-containing complex component 3	FLJ10038, ANKRD62, DDX54, GABPB2	1.30
BKN29023	CG9610	FBgn0003129	Pox meso	PAX4, PAX1, ARX, PAX8, PAX5, PAX6, P	1.30
BKN46240	CG5969	FBgn0036998	-	QPCT, QPCTL, VMA21	1.30
BKN34086	HDC07596		-	-	1.30
BKN27767	CG4247	FBgn0038307	mitochondrial ribosomal protein S10	MRPS10	1.30
BKN21387	CG5300	FBgn0032243	Kinesin-like protein at 31E	KIF21B, KIF7, KIF4A, KIF4B, KIF21A, KIF	1.30
BKN24399	CG14537	FBgn0031954	-	-	1.30
BKN29718	CG2302	FBgn0015519	nicotinic Acetylcholine Receptor alpha3	CHRNA7, CHRNB3, CHRNA5, CHRNA6,	1.30
BKN29341	CG4581	FBgn0025352	Thiolase	HADHB	1.30
BKN29815	CG8301	FBgn0037717	-	ZNF560, ZBTB41, ZNF782	1.30
BKN33078	CG7118	FBgn0035886	Jonah 66Ci	PROC, C1S, KLK3	1.30
BKN34060	HDC14891		-	-	1.30
BKN40622	CG45784	FBgn0058204	Myosin 81F	-	1.30
BKN29623	CG10481	FBgn0032827	-	XPR1	1.30
BKN28502	CG8841	FBgn0033713	-	HID1	1.30
BKN40020	CG43744	FBgn0085272	bruno 3	CELF3, CELF6, CELF2, CELF4, CELF1, C	1.30
BKN30880	CG12653	FBgn0000233	buttonhead	EGR1, ZNF526, SP7, SP8, EGR3, SP9, S	1.30
BKN32444	CG14968	FBgn0035431	-	LDLRAP1	1.29
BKN40656	CG8201	FBgn0026193	par-1	MARK2, MARK3, MARK4, MARK1, SIK3,	1.29
BKN24753	CG8172	FBgn0033362	-	PRSS21, CTRC, CTRL, PRSS48, TMPRS	1.29
BKN22505	CG4306	FBgn0036787	-	GGCT	1.29
BKN46603	CG32829	FBgn0052829	-	-	1.29
BKN22129	CG4972	FBgn0032217	-	NCLN	1.29
BKN22651	CG1665	FBgn0033451	-	MARC2, MARC1	1.29
BKN24717	CG9731	FBgn0037640	polychaetoid	TJP2, TJP1, TJP3	1.29
BKN25491	CG3508	FBgn0038251	Hexamethylene bisacetamide inducible	HEXIM1, HEXIM2	1.29
BKN29020	CG11824	FBgn0085379	Notopleural	CELA2A, TMPRSS13, CELA1, GZMA, GZ	1.29
BKN46587	CG43720	FBgn0032825	sickie	NAV1, NAV2, NAV3	1.29
BKN46006	CG10641	FBgn0032731	Swiprosin-1	EFHD2, EFHD1	1.29
BKN22438	CG3314	FBgn0014026	Ribosomal protein L7A	RPL7A	1.29
BKN23627	CG14528	FBgn0039611	-	MME, KEL, ECE1, ECEL1, MMEL1, ECE2	1.29
BKN33084	CG32985	FBgn0052985	-	-	1.29
BKN24690	CG13868	FBgn0034501	-	-	1.29
BKN28474	CG13213	FBgn0033609	F-box and leucine-rich repeat protein 6	FBXL19, LRRC29, KDM2A, FBXL4, KDM2	1.29
BKN29614	CG17272	FBgn0038830	-	CALN1, CALB1, OCM2, CALM2, CABP5,	1.29
BKN46586	CG12672	FBgn0030886	-	-	1.29
BKN40315	CG33717	FBgn0260458	Peptidoglycan recognition protein LD	PGLYRP2, PGLYRP4, PGLYRP1, PGLYR	1.29
BKN27926	CG9882	FBgn0034817	Arginine methyltransferase 7	PRMT7	1.29
BKN45590	CG3740	FBgn0023530	-	C19orf12	1.28
BKN29220	CG9852	FBgn0010340	upstream of RpII140	TIMMDC1	1.28
BKN30136	CG30320	FBgn0050320	-	-	1.28

BKN20900	CG17360	FBgn0037949	-	PLEKHM2	1.28
BKN20486	CG11305	FBgn0039631	Sirtuin 7	SIRT7, SIRT6	1.28
BKN42399	CR12798	FBgn0039987	-	-	1.28
BKN29380	CG10076	FBgn0003475	spire	SPIRE1, SPIRE2	1.28
BKN33135	CG1442	FBgn0039622	eukaryotic translation initiation factor 4E6	SESN3, SESN1, EIF4E3, EIF4E, EIF4E1B	1.28
BKN41807	CG12552	FBgn0040004	-	-	1.28
BKN22541	CG5429	FBgn0010709	Autophagy-related 6	BECN1, BECN2	1.28
BKN28547	CG9091	FBgn0030616	Ribosomal protein L37a	RPL37	1.28
BKN30223	CG12330	FBgn0035686	Cuticular protein 65Az	-	1.28
BKN20022	CG11796	FBgn0036992	-	HIP1R, HIP1, HPDL, HPD	1.28
BKN20016	CG7516	FBgn0028529	lethal (2) 34Fd	NOL10	1.28
BKN28933	CG17248	FBgn0013342	neuronal Synaptobrevin	VAMP2, VAMP8, VAMP3, VAMP4, VAMP[illegible]	1.28
BKN21997	CG11148	FBgn0039936	Gigyf	GIGYF1, GIGYF2	1.28
BKN28794	CG5701	FBgn0036980	Rho-related BTB domain containing	CDC42, RAC3, RHOBTB1, RHOD, RHOB[illegible]	1.28
BKN41927	CG8669	FBgn0000370	cryptocephal	ATF5, ATF4	1.28
BKN20242	CG3637	FBgn0025865	Cortactin	CTTN, HCLS1	1.27
BKN23565	CG31974	FBgn0051974	-	-	1.27
BKN20715	CG9646	FBgn0034184	-	KIAA0930	1.27
BKN41568	CG6090	FBgn0039406	Ribosomal protein L34a	RPL34	1.27
BKN31808	CG12279	FBgn0038080	-	TSTD3, TSTD1	1.27
BKN20756	CG6525	FBgn0082831	protein partner of snf	SETD1B, KMT2D, ASH1L, SETD2, PHF20	1.27
BKN28942	CG3186	FBgn0034967	eukaryotic translation elongation factor 5	EIF5A, EIF5A2, EIF5AL1	1.27
BKN27343	CG9023	FBgn0015872	Drip	AQP9, AQP7, AQP1, AQP10, MIP, FAM18	1.27
BKN28945	CG9344	FBgn0034564	-	LSM6	1.27
BKN41716	CG12856	FBgn0033966	-	-	1.27
BKN20849	CG8552	FBgn0031990	Phosphatidic Acid Phospholipase A1	SEC23IP, DDHD1, DDHD2	1.27
BKN29426	CG17521	FBgn0024733	Ribosomal protein L10	RPL10L, RPL10	1.27
BKN20008	CG8745	FBgn0036381	-	PHYKPL, ETNPPL	1.27
BKN28248	CG4111	FBgn0029785	Ribosomal protein L35	RPL35	1.27
BKN32704	CG1147	FBgn0037408	Neuropeptide F receptor	CH17-360D5.1, LOC105379861, NPY4R, [illegible]	1.27
BKN33377	CG15922	FBgn0040575	-	-	1.27
BKN21681	CG9474	FBgn0028401	Synaptosomal-associated protein 24kDa	SNAP47, SNAP23, SNAP25, SNAP29	1.27
BKN31260	CG9283	FBgn0036878	Cuticular protein 76Ba	-	1.27
BKN30570	CG14747	FBgn0259226	-	-	1.27
BKN30371	CG4385	FBgn0003310	Star	-	1.27
BKN29005	CG10055	FBgn0037482	-	SHQ1	1.27
BKN34483	HDC11104		-	-	1.27
BKN34049	HDC06597		-	-	1.27
BKN21657	CG6049	FBgn0037081	barricade	HTATSF1	1.27
BKN42049	CG32400	FBgn0020644	Lcp65Ab1	-	1.27
BKN25171	CG11927	FBgn0031638	-	-	1.27
BKN31991	CG15924	FBgn0085428	NADPH oxidase	NOX3, NOX4, NOX5, DUOX1, CYBB, DU[illegible]	1.27
BKN46017	CG9619	FBgn0036862	Glycogen binding subunit 76A	PPP1R3E, PPP1R3F, PPP1R3C, PPP1R3	1.26
BKN30167	CG11175	FBgn0034530	Reduction in Cnn dots 6	-	1.26
BKN20208	CG13570	FBgn0015544	spaghetti	RPAP3	1.26
BKN31176	CG12708	FBgn0030666	-	-	1.26
BKN22405	CG7530	FBgn0038256	-	PAQR3, ADIPOR1, PAQR8, ADIPOR2, PA	1.26
BKN45023	CG7281	FBgn0004597	Cyclin C	CCNC	1.26
BKN27388	CG6833	FBgn0036405	-	AEN, REXO4, ISG20, ISG20L2	1.26
BKN42313	CG41614	FBgn0085721	-	-	1.26
BKN27848	CG11586	FBgn0035520	-	ZMAT2	1.26
BKN40598	CR30220	FBgn0050220	transfer RNA:Glutamic acid-CTC 2-2	-	1.26
BKN28611	CG18492	FBgn0026323	TGF-beta activated kinase 1	DSTYK, RIPK3, MAP3K10, LRRK1, LRRK[illegible]	1.26
BKN22614	CG4673	FBgn0039348	Nuclear protein localization 4	NPLOC4	1.26
BKN30095	CG9427	FBgn0037721	-	IFI30	1.26
BKN20268	CG12082	FBgn0035402	Ubiquitin specific protease 5	USP5, USP13	1.26
BKN40546	CG14464	FBgn0033000	-	THAP7, THAP8, ARL14EP, ARL14EPL	1.26
BKN20165	CG11505	FBgn0035424	-	LARP4, LARP4B	1.26
BKN30497	CG3975	FBgn0027559	Pol32 polymerase delta subunit	POLD3	1.26
BKN30542	CG1561	FBgn0030317	-	-	1.26
BKN33114	CG8869	FBgn0031654	Jonah 25Bii	-	1.25
BKN29432	CG7434	FBgn0015288	Ribosomal protein L22	RPL22, RPL22L1	1.25

BKN32850	CG8193	FBgn0033367	Prophenoloxidase 2	-	1.25
BKN21293	CG4539	FBgn0010520	Bekka	FCF1	1.25
BKN22351	CG10306	FBgn0034654	eukaryotic translation initiation factor 3 subunit k	EIF3K	1.25
BKN20280	CG8411	FBgn0005695	germ cell-less	KLHL10, BTBD16, GMCL1	1.25
BKN31717	CG7991	FBgn0035260	-	-	1.25
BKN28400	CG9884	FBgn0011818	out at first	OAF	1.25
BKN24685	CG15227	FBgn0034554	-	CA5B, CA11, CA13, CA2, CA7, CA12, CA!	1.25
BKN41812	CG11100	FBgn0037207	Mesoderm-expressed 2	-	1.25
BKN33703	HDC05885	FBgn0085260	-	-	1.25
BKN29733	CG2916	FBgn0026361	Septin 5	SEPT7, SEPT11, SEPT14, SEPT5, SEPT2	1.25
BKN22499	CG5924	FBgn0032154	mitochondrial DNA helicase	POLN, TWNK	1.25
BKN30066	CG33251	FBgn0053251	-	-	1.25
BKN28534	CG9907	FBgn0003036	paralytic	SCN5A, CACNA1S, CACNA1A, SCN7A, S	1.25
BKN22518	CG12345	FBgn0015323	Choline acetyltransferase	CRAT, CPT1A, CHAT, CPT1B, CPT1C	1.25
BKN26937	CG12092	FBgn0031140	Niemann-Pick type C-1b	SCAP, NPC1L1, NPC1	1.25
BKN29191	CG7533	FBgn0036165	charybde	DDIT4, DDIT4L	1.25
BKN21384	CG8781	FBgn0033378	tsunagi	NCL, RBM8A	1.25
BKN26702	CG31861	FBgn0051861	-	-	1.25
BKN21655	CG10997	FBgn0030529	Chloride intracellular channel	CLIC6, CLIC1, CLIC2, CLIC5, CLIC3, CLI(	1.25
BKN31292	CG30269	FBgn0050269	-	LITAF, CDIP1	1.24
BKN22548	CG8144	FBgn0026188	pasilla	NOVA1, NOVA2	1.24
BKN40658	CG12547	FBgn0250830	-	NHLRC2	1.24
BKN23465	CG9377	FBgn0032507	-	F9, F10	1.24
BKN45947	CG1374	FBgn0003866	teashirt	TSHZ1, TSHZ3, TSHZ2	1.24
BKN28754	CG7424	FBgn0031980	Ribosomal protein L36A	RPL36AL, RPL36A, RPL36A-HNRNPH2	1.24
BKN22031	CG5330	FBgn0015268	Nucleosome assembly protein 1	NAP1L3, NAP1L2, NAP1L4, NAP1L1, NAF	1.24
BKN21404	CG1882	FBgn0033226	-	ABHD4, ABHD5, EPHX3	1.24
BKN22782	CG3297	FBgn0002778	minidiscs	SLC7A5, SLC7A6, SLC7A13, SLC7A14, S	1.24
BKN28802	CG14745	FBgn0043575	Peptidoglycan recognition protein SC2	PGLYRP2, PGLYRP4, PGLYRP3, PGLYR	1.24
BKN28570	CG4428	FBgn0031298	Autophagy-related 4a	ATG4A, ATG4D, ATG4B, ATG4C	1.24
BKN46607	CG13239	FBgn0037197	-	-	1.24
BKN29068	CG2471	FBgn0030357	Sclp	LRRC20	1.24
BKN26648	CG33770	FBgn0053770	-	-	1.24
BKN22424	CG11301	FBgn0034726	Mesoderm-expressed 4	NFYC, POLE4, CHRAC1	1.24
BKN40402	CR42890	FBgn0046822	mir-3 stem loop	-	1.24
BKN33104	CG3960	FBgn0085446	-	SMTNL1, SMTN, SMTNL2, SRRM3, SPEC	1.24
BKN27686	CG2701	FBgn0024973	-	ASCC2	1.24
BKN28835	CG4905	FBgn0034135	Syntrophin-like 2	SNTG2, SNTG1	1.24
BKN25885	CG5873	FBgn0038511	Curly Su	EPX, LPO, TPO, PTGS2, MPO, PXDNL, P	1.24
BKN23031	CG10110	FBgn0024698	Cleavage and polyadenylation specificity factor 160	CPSF1	1.23
BKN20303	CG8194	FBgn0010406	Ribonuclease X25	RNASET2	1.23
BKN28173	CG12220	FBgn0039835	mitochondrial ribosomal protein L32	MRPL32	1.23
BKN30923	CG15628	FBgn0031632	-	GLYATL3	1.23
BKN27003	CG31367	FBgn0051367	-	TCERG1L, TCERG1, ARHGAP5	1.23
BKN31143	CG14248	FBgn0250908	beat-VII	CADM2	1.23
BKN27869	CG8784	FBgn0038140	Pyrokinin 2 receptor 1	NTSR2, GPR39, NMUR2, GPR148, NMUF	1.23
BKN33402	HDC08633		-	-	1.23
BKN22662	CG2984	FBgn0022768	Protein phosphatase 2C	ILKAP, PP2D1, PPM1F, PPM1L, PPM1E,	1.23
BKN28176	CG7726	FBgn0013325	Ribosomal protein L11	RPL11	1.23
BKN24244	CG1866	FBgn0039581	Moca-cyp	PPIG, RGPD4, PPIAL4G, NKTR, RGPD5,	1.23
BKN22875	CG32076	FBgn0052076	Alpha 3 glucosyltransferase	ALG10, ALG10B	1.23
BKN42482	CG34439	FBgn0085468	NADH dehydrogenase (ubiquinone) MWFE subunit	NDUFA1, NCAPG	1.23
BKN28567	CG7902	FBgn0004862	bagpipe	NKX2-8, NKX2-4, NKX2-5, NKX3-2, NKX2	1.23
BKN22520	CG6920	FBgn0002906	Bloom syndrome helicase	BLM, RECQL5, WRN, RECQL	1.23
BKN27510	CG10664	FBgn0032833	Cytochrome c oxidase subunit 4	COX4I1, COX4I2	1.23
BKN26643	CG30015	FBgn0050015	-	-	1.23
BKN31213	CG13681	FBgn0035832	Ectoderm-expressed 4	SARM1, TMEM199	1.23
BKN24757	CG32119	FBgn0052119	-	-	1.23
BKN21643	CG15651	FBgn0034567	-	FKRP	1.23
BKN30056	CG6272	FBgn0036126	Inverted repeat binding protein 18 kDa	CEBPB, TEF, CEBPG, DBP, HLF, CEBPA	1.23
BKN46656	CG42551	FBgn0260724	La related protein	LARP1B, LARP1	1.23
BKN31638	CG31191	FBgn0051191	-	-	1.23

BKN22441	CG5116	FBgn0039339	-	GTPBP6	1.23
BKN30101	CG15816	FBgn0030866	-	RASAL1, RASA1, DAB2IP, RASAL2, RAS/	1.23
BKN46267	CG9760	FBgn0036259	-	-	1.23
BKN40958	CG7463	FBgn0036504	yellow-k	-	1.23
BKN22544	CG5197	FBgn0085487	-	KLK10, KLK15, KLK9, CTSG, GZMB, KLK	1.23
BKN33119	CG10677	FBgn0035596	-	-	1.23
BKN26060	CG8484	FBgn0037751	matotopetli	ZNF543, ZBTB24, ZNF331, ZNF785	1.23
BKN29002	CG4204	FBgn0023212	Elongin B	TCEB2	1.23
BKN29103	CG17332	FBgn0027779	Vacuolar H[+]-ATPase SFD subunit	ATP6V1H	1.23
BKN26293	CG3618	FBgn0037028	-	FAM234B, FAM234A	1.23
BKN25757	CG13724	FBgn0036707	-	-	1.22
BKN25222	CG31998	FBgn0051998	-	-	1.22
BKN45386	CG18362	FBgn0032940	Mondo	MLXIPL, MLXIP	1.22
BKN30483	CG9728	FBgn0039780	prolyl-4-hydroxylase-alpha NE1	P4HTM, P4HA3, P4HA2, P4HA1	1.22
BKN27078	CG31348	FBgn0250910	Octopamine beta3 receptor	TAAR8, HTR4, TAAR6, TAAR9, TAAR1, T	1.22
BKN29739	CG31075	FBgn0051075	-	ALDH1A1, ALDH9A1, ALDH8A1, ALDH2, /	1.22
BKN28315	CG4084	FBgn0011297	lethal (2) neighbor of tid	ALG3	1.22
BKN30200	CG15582	FBgn0046878	Odorant-binding protein 83cd	-	1.22
BKN26408	CG17625	FBgn0039002	-	SASH1, INPPL1, SAMSN1, SAMD5, SASH	1.22
BKN24452	CG6735	FBgn0086690	Pericentrin-like protein	PCNT, AKAP9	1.22
BKN30544	CG7502	FBgn0259169	Rho GTPase activating protein at 18B	ARHGAP29, SYDE1, RACGAP1, ARHGAF	1.22
BKN22059	CG12325	FBgn0033557	-	LOC102724159, PWP2	1.22
BKN27863	CG12740	FBgn0035422	Ribosomal protein L28	RPL28	1.22
BKN41376	CG12491	FBgn0034900	-	-	1.22
